# Synthesis and photophysical studies of a multivalent photoreactive Ru^II^-calix[4]arene complex bearing RGD-containing cyclopentapeptides

**DOI:** 10.3762/bjoc.14.150

**Published:** 2018-07-16

**Authors:** Sofia Kajouj, Lionel Marcelis, Alice Mattiuzzi, Adrien Grassin, Damien Dufour, Pierre Van Antwerpen, Didier Boturyn, Eric Defrancq, Mathieu Surin, Julien De Winter, Pascal Gerbaux, Ivan Jabin, Cécile Moucheron

**Affiliations:** 1 Laboratoire de Chimie Organique et Photochimie, Université libre de Bruxelles, Avenue F.D. Roosevelt 50, CP 160/08, 1050 Bruxelles, Belgium; 2 Engineering of Molecular NanoSystems, Ecole Polytechnique de Bruxelles, Université libre de Bruxelles (ULB), Avenue F.D. Roosevelt 50, CP165/64, B-1050 Brussels, Belgium; 3 Laboratoire de Chimie Organique, Université libre de Bruxelles, Avenue F.D. Roosevelt 50, CP 160/06, 1050 Bruxelles, Belgium; 4 Université Grenoble Alpes, Département de Chimie Moléculaire UMR CNRS 5250, CS 40700, 38058 Grenoble Cedex 09, France; 5 Analytical Platform of the Faculty of Pharmacy, Université libre de Bruxelles, Boulevard du Triomphe, Campus de la Plaine, CP205/05, 1050 Bruxelles, Belgium; 6 Laboratory for Chemistry of Novel Materials, Center for Innovation and Research in Materials and Polymers, University of Mons – UMONS, 20, Place du Parc, B-7000 Mons, Belgium; 7 Organic synthesis and Mass Spectrometry Laboratory, University of Mons - UMONS, Place du Parc 23, B-7000 Mons, Belgium

**Keywords:** anticancer drug, calixarene, cell targeting, RGD peptide, ruthenium complex

## Abstract

Photoactive ruthenium-based complexes are actively studied for their biological applications as potential theragnostic agents against cancer. One major issue of these inorganic complexes is to penetrate inside cells in order to fulfil their function, either sensing the internal cell environment or exert a photocytotoxic activity. The use of lipophilic ligands allows the corresponding ruthenium complexes to passively diffuse inside cells but limits their structural and photophysical properties. Moreover, this strategy does not provide any cell selectivity. This limitation is also faced by complexes anchored on cell-penetrating peptides. In order to provide a selective cell targeting, we developed a multivalent system composed of a photoreactive ruthenium(II) complex tethered to a calix[4]arene platform bearing multiple RGD-containing cyclopentapeptides. Extensive photophysical and photochemical characterizations of this Ru(II)–calixarene conjugate as well as the study of its photoreactivity in the presence of guanosine monophosphate have been achieved. The results show that the ruthenium complex should be able to perform efficiently its photoinduced cytotoxic activity, once incorporated into targeted cancer cells thanks to the multivalent platform.

## Introduction

Long-living luminescent polyazaaromatic ruthenium(II) complexes are intensively studied in a biological context, in particular (i) for their ability to sense their environment and (ii) for their photoreactivity towards relevant biological targets [1–4]. Sensors for biological species are mostly based on complexes bearing the well-known dppz ligand (dppz = dipyrido[3,2-*a*:2’,3’-*c*]phenazine) and its derivatives. J. K. Barton et al. demonstrated in 1990 that [Ru(bpy)_2_(dppz)]^2+^ behaves as a light-switch for DNA [5]: this complex is not luminescent in water but upon intercalation within the DNA base pairs stack, the complex luminescence is restored. Derivatives of [Ru(bpy)_2_(dppz)]^2+^ and complexes bearing similar aromatic planar ligands were developed to probe specific sites of DNA, such as mismatches [6–8], abasic sites [9] or G-quadruplexes [10–11]. Aside photosensors, photoreactive complexes able to damage biological targets were also developed. These complexes are mainly used to induce damages in cancerous cells upon light irradiation. Two types of photooxidative damages can be induced: (i) by photosensitization of singlet oxygen and subsequent generation of highly reactive oxygen species (ROS) (type I photosensitization) or (ii) by direct oxidative electron transfer to biological molecules such as DNA or amino acids (type II photosensitization). In particular, it was shown that Ru^II^ complexes containing at least two highly π-deficient polyazaaromatic ligands such as 1,4,5,8-tetraazaphenanthrene (TAP) [12–14] or 1,4,5,8,9,12-hexaazatriphenylene (HAT) [15] are able to oxidize the guanine base (G) of DNA or the tryptophan (Trp) amino acid residue through a photoinduced electron-transfer (PET) process [16–19]. Interestingly, the two radical species generated by this PET can recombine to form a covalent photoadduct [20–22]. When this photoadduct is formed with the guanine base, the activity of enzymes such as RNA polymerase or endonuclease is inhibited in vitro at the level of the photoadduct [23–24]. In order to target a specific DNA sequence, photoreactive Ru^II^ complexes have been anchored to specific antisense oligonucleotides to inhibit the expression of the complementary targeted genes under illumination [25–26]. This photoinduced gene-silencing strategy has been proven to be also efficient in living cells [27–28], paving the way for the use of photoactivable Ru^II^ complexes as photocontrolled anticancer therapeutic agents.

Despite their interesting photochemical properties, photoreactive Ru^II^ complexes have shown low cell-penetration efficiency, preventing their direct use in biological applications. More lipophilic ligands such as bathophenanthroline and modified dppz were developed and the internalization of the corresponding Ru^II^ complexes was demonstrated [29–32]. These complexes are however not photoreactive due to the absence of π-deficient ligands. More recently, Ru^II^ complexes bearing two modified TAP ligands with highly lipophilic moieties were reported [33]. These compounds are able to enter the cells and photoinduce caspase-dependent and reactive-oxygen-species-dependent apoptosis. Another strategy for the design of cell penetrating photoreactive Ru^II^ complexes consists of tethering the complex to a vector that allows a cellular uptake. In this context, Os^II^, Rh^III^ and Ru^II^ complexes were anchored to cell penetrating peptides (CPP) such as polyarginine [34–37]. The tethering of a photoreactive Ru^II^ complex on the *transactivating transcriptional activator* (TAT) peptide was also reported and it was shown that the corresponding Ru^II^ conjugate could be internalized inside HeLa cells without any modification of the photochemical properties of the complex [38].

It should be noted that modifications of ligands to make the resulting complexes more lipophilic or the conjugation of a complex to a CPP do not provide any control on the way these complexes will be internalized by cells and prevent thus any targeting of malignant cells over healthy ones. The next step in the development of phototherapeutic agents based on polyazaaromatic Ru^II^ complexes is thus the specific targeting of cancerous cells. In this regard, α_v_β_3_ integrin represents an interesting target as this membrane receptor is overexpressed in the endothelial cells of neoangiogenic vessels and in several human tumor cells [39–40]. It is well known that RGD-containing oligopeptides (RGD = Arg-Gly-Asp tripeptide pattern) bind selectively to α_v_β_3_ integrin with a high affinity and a very high selectivity [41–43]. As multivalency enhances the binding strength of a ligand to its receptor [44–46], clustered RGD-containing compounds were developed and were shown to exhibit attractive biological properties for the imaging of tumors [47–50] and for the targeted drug delivery [51–53].

In the course of designing phototherapeutic agents that could specifically target cancerous cells, we envisaged to graft a photoreactive Ru^II^ complex on a multivalent platform decorated with multiple RGD-containing cyclopentapeptides. A calix[4]arene moiety was chosen as the multivalent platform as this rigid macrocycle displays two distinct faces that can be selectively functionalized [54–56]. It is noteworthy that the calix[4]arene skeleton has been already exploited for the development of multivalent glyco- and peptidocalixarenes that can be recognized by cell-membrane receptors [57–59] and of calixarene derivatives able to specifically target membrane proteins involved in the angiogenesis process [60]. Furthermore, the use of calixarenes for biological applications is the subject of intensive researches. They are indeed exploited in various areas such as surface recognition, structural mimes or membrane receptor inhibition [61–63], and it was also shown that calixarenes themselves display antibacterial, antiviral, and anticancer properties [64].

**Figure 1:**
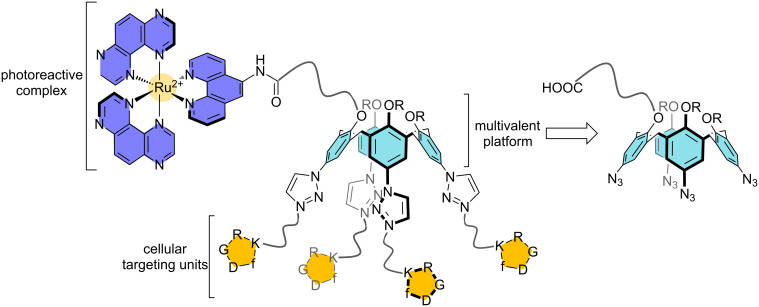
Targeted multivalent phototherapeutic agent and its calix[4]arene-based precursor. RGD = Arg–Gly–Asp residues, f = D-Phe residue.

Herein, we describe the synthesis of a multivalent phototherapeutic agent designed in order to specifically target membrane receptors involved in the angiogenesis process. The multivalent system is composed of a photoreactive [Ru(TAP)_2_phen]^2+^ complex tethered to a calix[4]arene platform bearing four c-[RGDfK] moieties [65] (Figure 1). Before studying this conjugate in vitro, it was first mandatory to check that the photochemistry of the Ru^II^ complex was not altered by the presence of the targeting platform. The photophysical properties of this Ru^II^–calixarene conjugate were thus examined and compared to those of the reference complex [Ru(TAP)_2_phen]^2+^.

## Results and Discussion

### Synthesis of Ru^II^-calixarene conjugate **9**

**Scheme 1:**
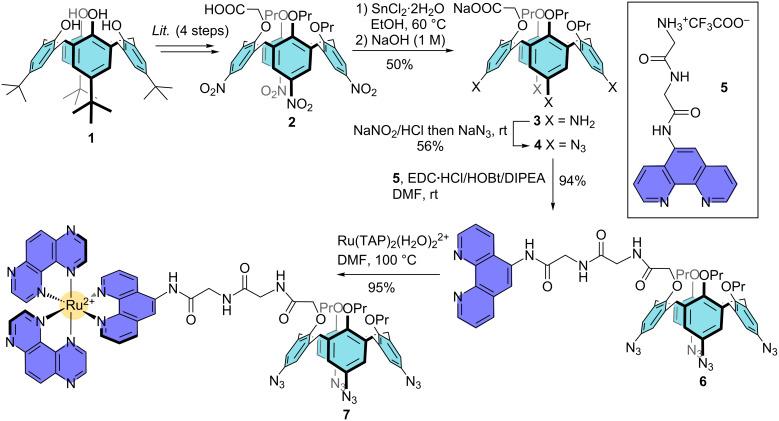
Synthesis of Ru^II^-calix[4]arene complex **7**.

For the synthesis of the target multivalent system, the strategy relies on the anchoring i) of the photoreactive [Ru(TAP)_2_phen]^2+^ complex on the calix[4]arene small rim through a peptide-type coupling and ii) of the four c-[RGDfK] moieties on the opposite rim through a copper-catalyzed azide–alkyne cycloaddition (CuAAC) [66–68] (Figure 1). It was thus necessary to block the calix[4]arene skeleton in the cone conformation and to functionalize separately the two distinct rims (Scheme 1). Firstly, known calixarene **2** with an appending carboxylate arm on the small rim was synthesized from commercial *p*-*tert-*butylcalix[4]arene **1** according to a four-step sequence [69]. Note that propyl groups were chosen for the modification of the small rim because these groups are the smallest possible for blocking the oxygen-through-the-annulus rotation of the aromatic units [70]. The nitro groups of **2** were then reduced using SnCl_2_·2H_2_O in ethanol, affording tetra-amino compound **3** [69] in 50% yield. Diazotation followed by nucleophilic substitution with sodium azide gave the desired tetra-azido compound **4** in 56% overall yield from **3**. It is noteworthy that the introduction of the azido groups on the calix[4]arene scaffold was clearly confirmed by the presence of an intense band at 2108 cm^−1^ in the IR spectrum of **4**. Phenanthroline derivative **5** was synthesized from 5-glycinamido-1,10-phenanthroline in a two-step sequence consisting of a peptide-type coupling reaction with a Boc-protected glycine *N*-hydrosuccinimide ester followed by the deprotection of the amino group (see Supporting Information File 1) [71]. Different coupling agents (DCC/HOBt, EDC·HCl/HOBt, PyBOP) and conditions were then tested for the peptide-type coupling reaction between calix[4]arene **4** and phenanthroline derivative **5**. The use of an excess of **5** (2 equiv) in the presence of EDC·HCl and HOBt in DMF at room temperature led to the best yield and the easiest purification process. Under these optimal conditions, the desired compound **6** was isolated in a high 94% yield. Finally, the reaction between [Ru(TAP)_2_(H_2_O)_2_]^2+^ and **6** in DMF at 100 °C gave the Ru^II^-calix[4]arene complex **7** in 95% yield after C18 reversed-phase silica gel column chromatographic purification. Complex **7** was fully characterized by 1D and 2D NMR spectroscopy in CD_3_CN at 600 MHz. In accordance with the presence for the chiral Ru(TAP)_2_phen moiety, the ^1^H NMR spectrum of **7** is characteristic of a *C*_1_ symmetrical compound as all the protons belonging to the ArH, ArCH_2_ and OPr group are differentiated. Moreover, complex **7** was also characterized by high-resolution mass spectrometry (HRMS). The ESI mass spectrum displays two intense signals at *m*/*z* 764.736 and *m*/*z* 1642.456 that are attributed respectively to the doubly charged **7^2+^** and singly charged [**7** + CF_3_COO^−^]**^+^** by comparison between the experimental and theoretical isotope distributions (see Supporting Information File 1).

**Scheme 2:**
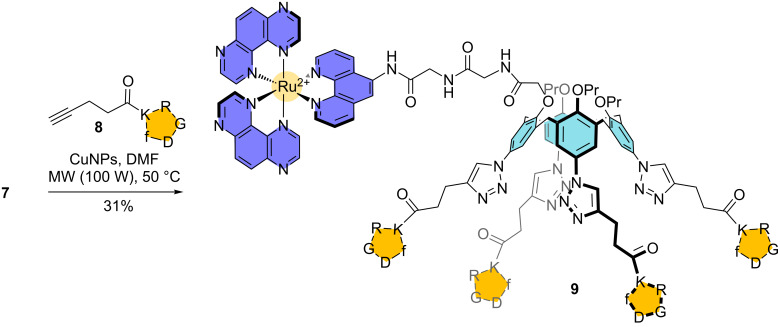
Synthesis of Ru^II^-calix[4]arene-[c-(RGDfK)]_4_ conjugate **9**.

With Ru^II^-calix[4]arene complex **7** in hands, we next moved to the introduction of the cellular targeting units on the large rim through copper-catalyzed azide–alkyne cycloaddition (CuAAC). Note that the triazole moieties that would result from such a cycloaddition are known to be stable towards hydrolysis and protease, which allows their use in a biological environment [72]. For the CuAAC, the use of Cu^I^-generated in situ from a mixture of CuSO_4_·5H_2_O and sodium ascorbate is often reported in the field of calixarene chemistry [66,73–78]. Unfortunately, this methodology led to poor yields and a lack of reproducibility in the case of calixarene **7** and c-[RGDfK]-alkyne **8**, even when a microwave heating was used. We then evaluated the use of copper nanoparticles (CuNPs), as these nanomaterials are known to catalyze efficiently a wide range of organic reactions and notably the azide–alkyne cycloaddition [79]. Calixarene **7** was reacted with a slight excess (5 equiv) of cyclopeptide **8** in the presence of CuNPs and the mixture was heated by microwave (100 W) at 50 °C for 1 hour. The use of CuNPs greatly facilitated the monitoring of the reaction and the work-up, as these nanomaterials being easily removed from the crude mixture by simple centrifugation. To our delight, [Ru(TAP)_2_phen]^2+^-calix[4]arene-[c-(RGDfK)]_4_ conjugate **9** was isolated in 31% yield after purification by semi-preparative RP-HPLC (Scheme 2). The successful synthesis and purification of conjugate **9** was also confirmed by HRMS. Indeed, the ESI mass spectrum features several peaks corresponding to characteristic ions of different charge states at *m*/*z* 1421.577 (3+), 1066.434 (4+) and 853.556 (5+) that are attributed to [**9** + H]^3+^, [**9** + 2H]^4+^ and [**9** + 3H]^5+^ by comparison between the experimental and theoretical isotope distributions (see Supporting Information File 1).

**Figure 2:**
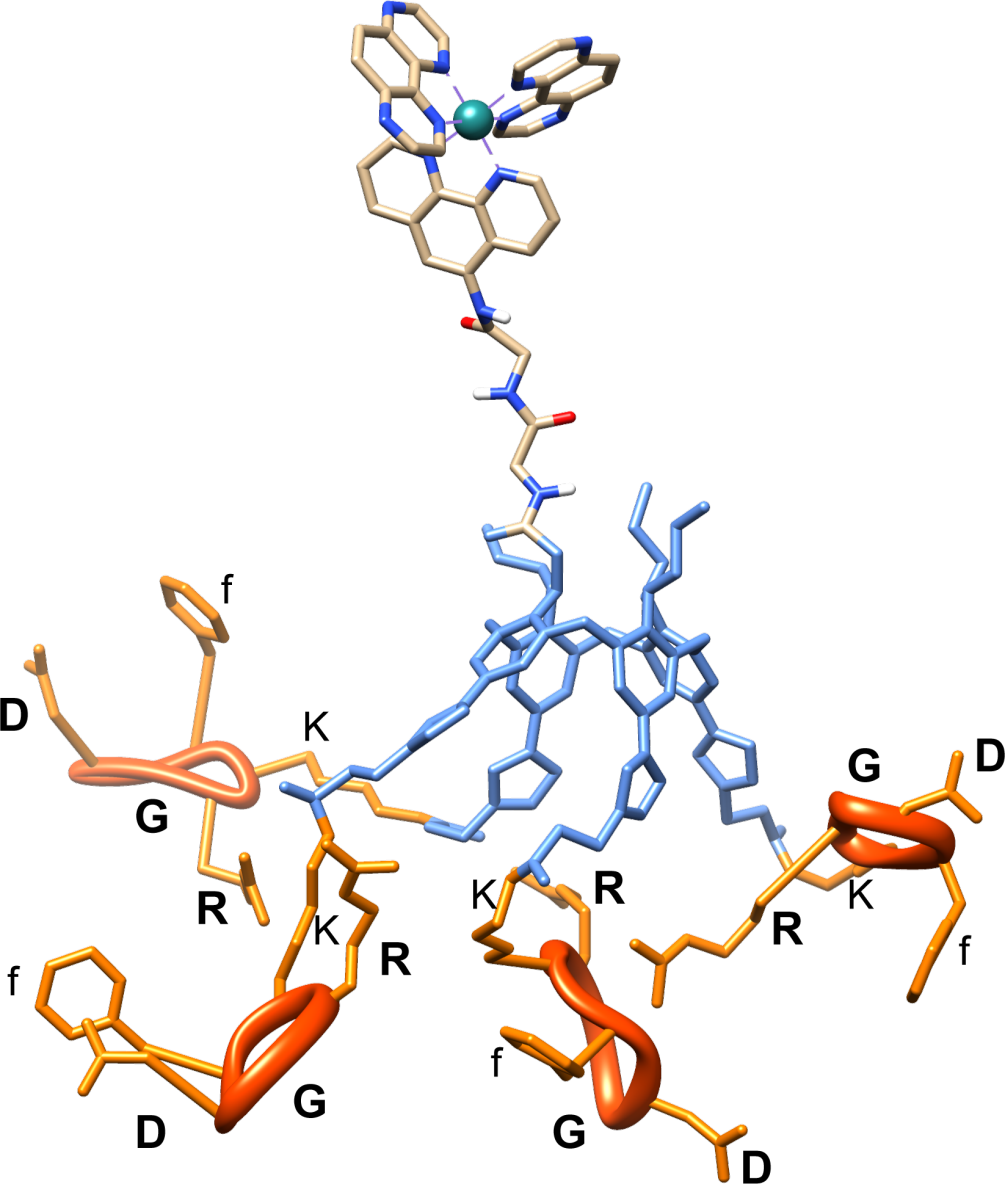
MD snapshot showing an optimized model of conjugate **9**. RGDfK units are depicted in orange ribbons, the calixarene is in blue and the Ru complex is colored by atom type.

Molecular modeling simulations were carried out to provide insights into the size and morphology of conjugate **9**. An optimized geometry is presented in Figure 2, as issued from a molecular dynamics (MD) simulations. The ruthenium complex and the RGD units are spatially well-separated thanks to their grafting on opposite faces of the rigid calixarene-based platform. In this conformation, the distances between the Ru atom and each of the nearest carbon atoms of RGDfK units exceed 30 Å. Along the MD simulations, we noticed that the Ru complex remained far from the cyclic pentapeptides. This is due to the fact that the linkers of each arm are smaller than the size of the calixarene platform, preventing contacts between the Ru complex and the RGDfK units. The global structure has an average radius of gyration *R*_g_ of 1.25 nm ± 0.1 nm. Noteworthy, the distance between the RGDfK units largely varies along the MD simulations, ranging from 10 Å to 24 Å (average at 17 Å), as estimated from the distance between equivalent carbon atoms crossing the linker and the cyclic pentapeptides. This large variation in the distance is due to the flexibility of the linkers between the calixarene platform and the RGDfK units, together with the many possibilities of H-bonding between: (i) oxygen atoms at C=O in the linker and the hydrogen atoms of (N–H) of arginine of a neighboring ‘arm’; (ii) H-bonds between arginine terminal N–H and C=O of the peptide bond of phenylalanine of an adjacent cyclic pentapeptide (see Supporting Information File 1), yielding adjacent cyclic pentapeptides in close proximity for a large set of conformations.

This separation between the Ru complex and the cyclic peptides by the calixarene should be an advantage by preventing any negative effect of the RGD peptidic units on the photochemistry of the complex and, alternatively, prevents any influence of the complex on the affinity of the RGD patterns to interact with the targeted integrins. However, the possible H-bonding interactions between neighboring RGD units could be a drawback in view of the accessibility of the arginine groups to interact with the integrins.

### Photophysical properties of Ru^II^-calixarene conjugate **9**

**Figure 3:**
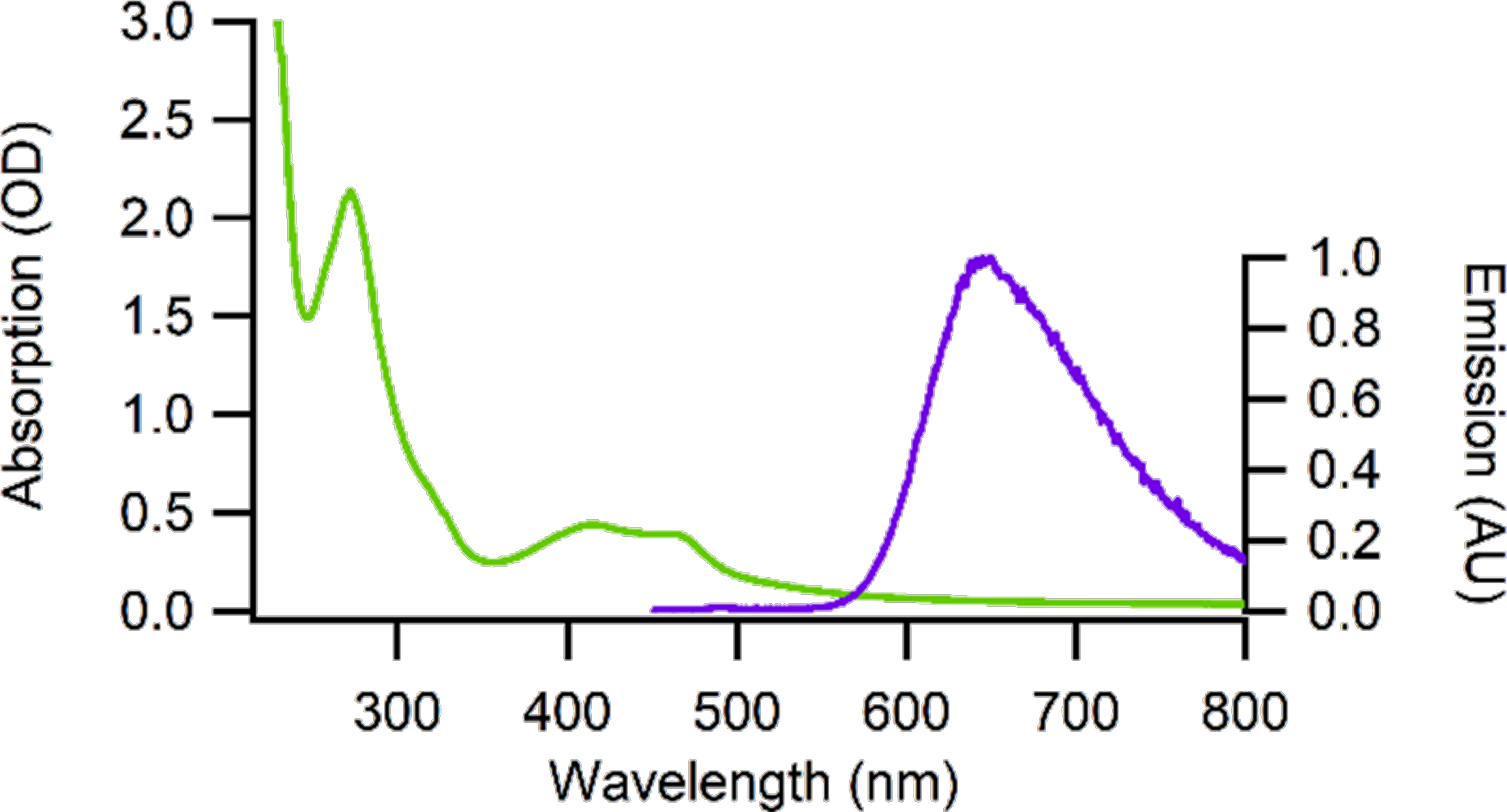
Absorption and emission spectra of Ru^II^-calix[4]arene-[c-(RGDfK)]_4_ conjugate **9** in water.

The absorption and emission spectra of Ru-calix(RGD)_4_ conjugate **9** (as its CF_3_COO^−^ salt) were recorded in water at room temperature (Figure 3). These spectroscopic data are gathered in Table 1 with the ones of the free [Ru(TAP)_2_phen]^2+^ complex for comparison purpose.

**Table 1:** Photophysical properties of conjugate **9** and [Ru(TAP)_2_phen]^2+^ in water.

Complex	λ^abs^ (nm)	λ^em^ (nm)	Φ^Air a,b^	Φ^Ar a,b^	τ_av_^Air b,c^ (ns)	τ_av_^Ar b,c^ (ns)

[Ru(TAP)_2_phen]^2+^	231, 272, 412, 464	645	0.029	0.055	714	891
conjugate **9**	274, 416, 458	645	0.025	0.044	901	1087

^a^Photoluminescence quantum yields are determined by comparison with [Ru(bpy)_3_]^2+^. Errors on Φ estimated to <20%. ^b^Measurement with 5% DMSO. ^c^Errors on lifetime estimated to 15%.

Conjugate **9** exhibits absorption bands at 416 and 458 nm that corresponds to dπ(Ru)–π*(phen/TAP) metal-to-ligand charge transitions (MLCT) similarly to what is observed for the untethered [Ru(TAP)_2_phen]^2+^ complex (MLCT bands at 412 and 464 nm). The presence of the calix[4]arene platform has thus no impact on the visible part of the spectrum. The influence of the calixarene moiety is however visible in the UV region of the spectrum (around 200 nm) where the absorption bands are more intense. This increase is due to the contribution of the peptidic subunits of the RGD moieties and of the aromatic units of the calixarene. It should be noted that the absorption at wavelengths longer than 550 nm does not go perfectly down to zero. This phenomenon is likely due to some light scattering caused by the presence of some small aggregates in solution. It appears that conjugate **9** is not completely soluble in pure water despite the presence of the charged Ru^II^ complex and the peptidic moieties on the calix[4]arene scaffold. Fortunately, these small aggregates totally disappeared when only 5% of DMSO was added to the medium [80].

The photoluminescence emission originating from the ^3^MLCT state is centered at 645 nm for both conjugate **9** and reference [Ru(TAP)_2_phen]^2+^complex. We measured the luminescence lifetime and determined the quantum yield of luminescence under air and argon atmosphere for conjugate **9** and reference [Ru(TAP)_2_phen]^2+^ in water with 5% DMSO in order to avoid any formation of aggregates. The data gathered in Table 1 clearly indicate that the tethering of the [Ru(TAP)_2_phen]^2+^ complex onto the calixarene platform does not induce any modification of the photophysical properties of the complex. In order to rule out any intramolecular quenching processes, control experiments were realized with the complex grafted onto the unmodified calixarene (conjugate **7**) in the presence of free cyclic pentapeptide units c-[RGDfK] **8** (see Supporting Information File 1). No modification of the luminescence by intermolecular quenching was observed, confirming the absence of internal quenching in the conjugate **9**.

### Photoreactivity of Ru^II^-calixarene conjugate **9**

**Figure 4:**
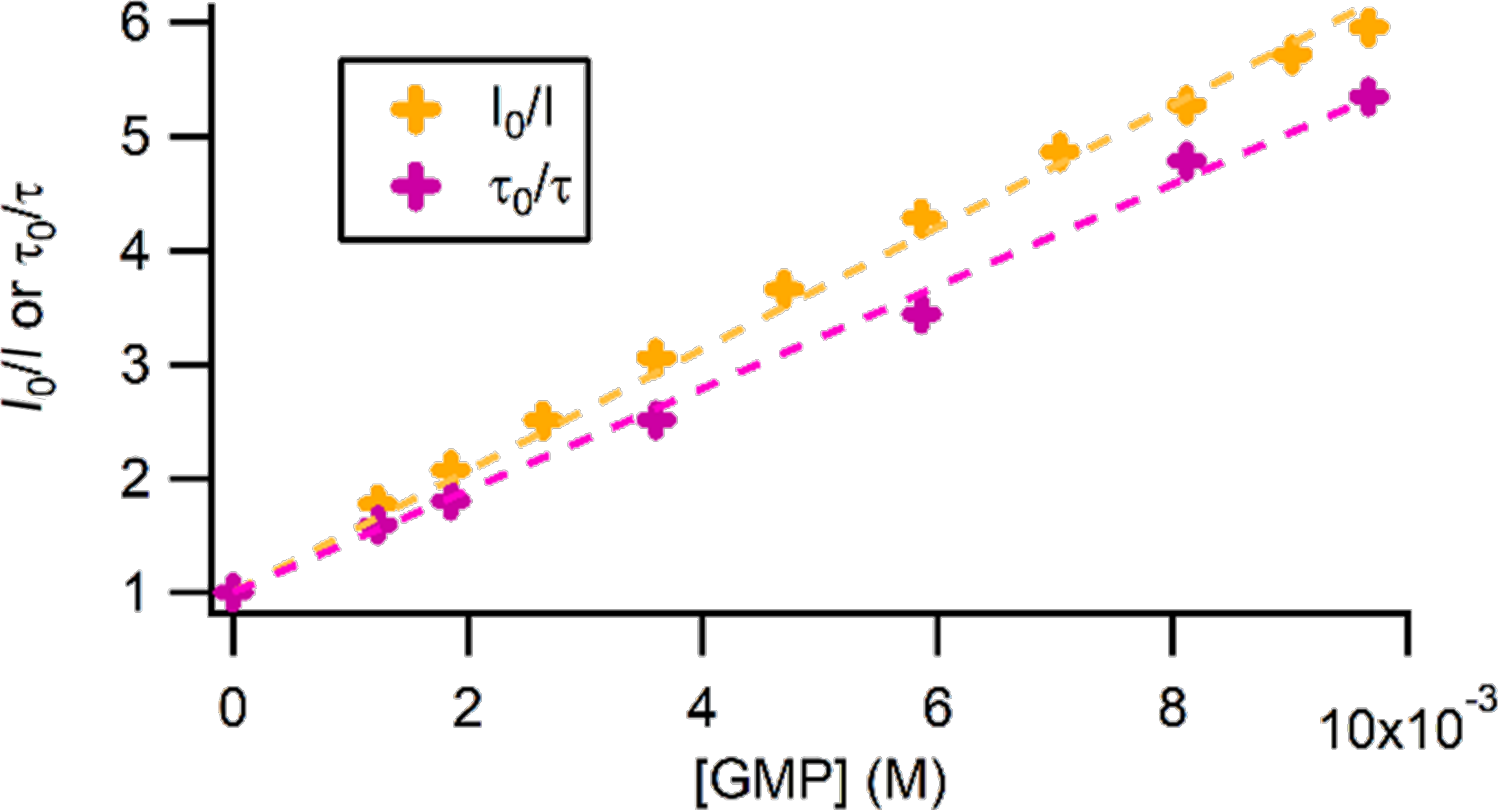
Luminescence intensity and excited state lifetime of conjugate **9** in the presence of GMP measured in 10 mM Tris·HCl buffer at pH 7.0.

The photoreactivity of Ru-TAP complexes is based on their ability to induce direct oxidation of guanine upon light excitation. In order to confirm that the tethering onto the calixarene platform does not impede the conjugated complex to photoreact with its biological target, we measured the evolution of the luminescence intensity and the excited state lifetime of conjugate **9** as function of the concentration of guanosine monophosphate (GMP, Figure 4).

**Figure 5:**
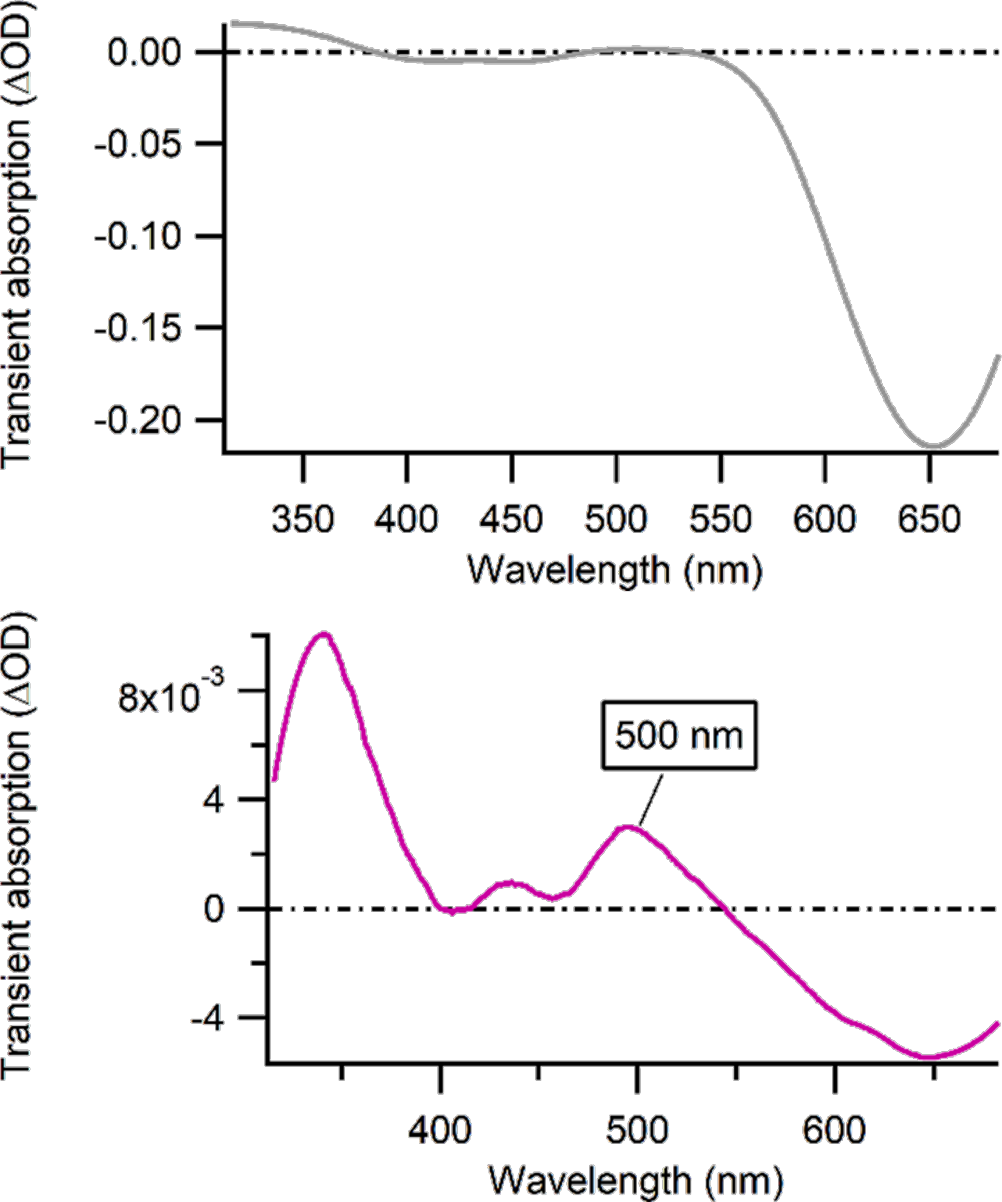
Transient absorption spectra of Ru^II^-calix[4]arene-[c-(RGDfK)]_4_ conjugate **9** (in 10 mM Tris·HCl buffer at pH 7.0) measured 500 ns after the laser pulse (gray, top) and 1 µs after the laser pulse in the presence of 10 mM GMP (purple, bottom).

Stern–Volmer analyses indicate that a dynamic quenching is occurring, with a quenching rate close to the diffusion limit (*k*_Q_ = 5.6 10^8^ M^−1^s^−1^ in intensity and *k*_Q_ = 5.3 10^8^ M^−1^s^−1^ in lifetime). This quenching of the luminescence of conjugate **9** in the presence of GMP reveals that a photoinduced electron transfer can take place between the excited complex and the guanine moiety, which could give rise to the formation of a photoadduct from the recombination of the monoreduced complex and the radical guanine generated after the photoinduced electron transfer (PET). In order to confirm the occurrence of PET, transient absorption measurements with conjugate **9** were performed in the absence and in the presence of GMP. The recorded transient absorption spectra are presented in Figure 5. In absence of GMP, the transient absorption spectrum of conjugate **9** is dominated by the luminescence, the ground state bleaching and some excited state absorption around 340 nm whereas in the presence of GMP a positive transient signal can be observed around 500 nm on a long time scale. This transient is specific of a monoreduced Ru^II^-TAP^•−^ species [81], confirming that a PET occurs.

**Figure 6:**
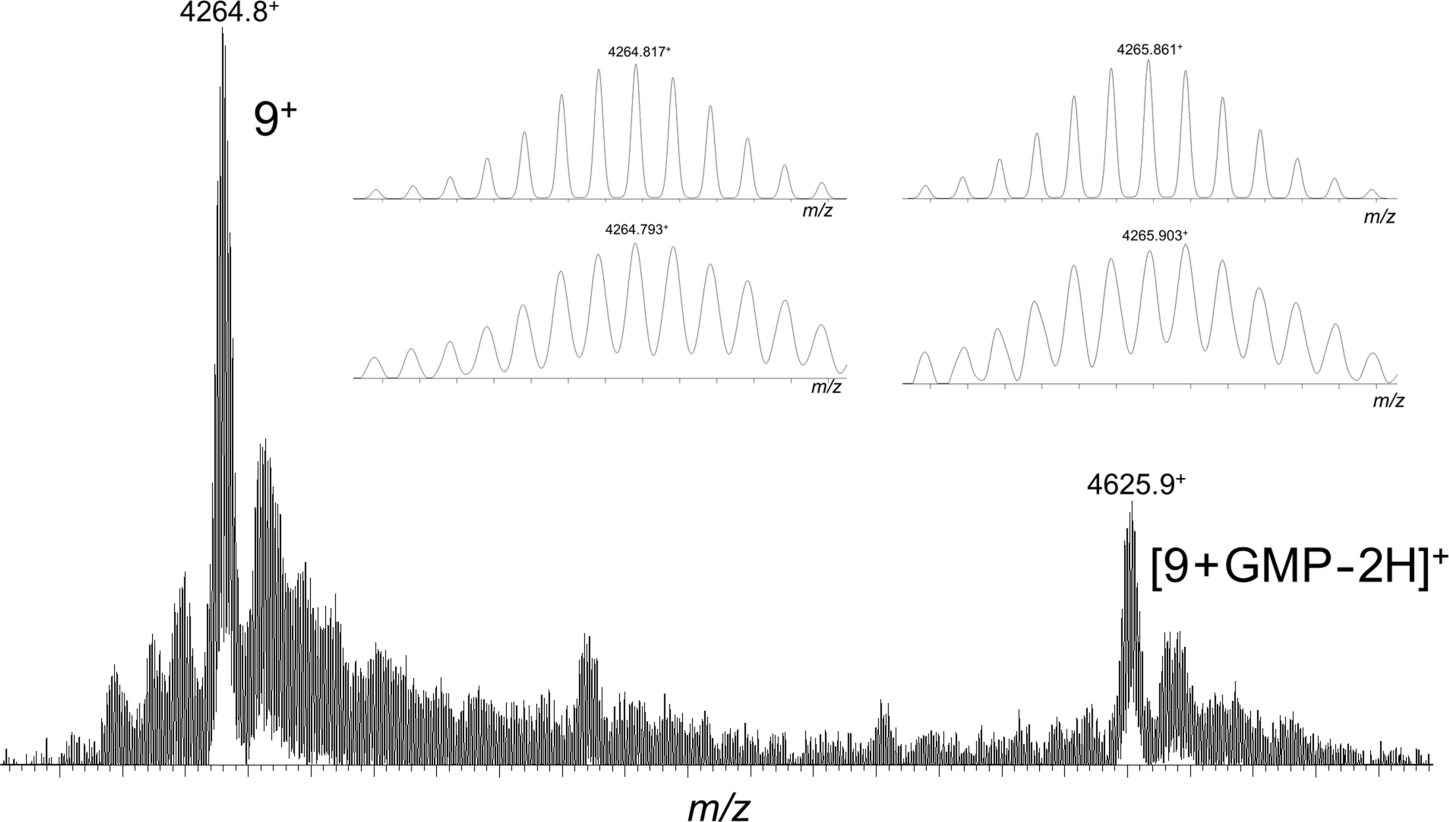
MALDI–MS analysis of a solution containing conjugate **9** and GMP after continuous light irradiation. In the inset, the experimental (bottom) and theoretical (top) isotope distributions are compared for both **9**^+^ and [**9** + GMP − 2H]^+^ ions.

To verify if a photoadduct can be obtained between the complex anchored on the calixarene platform and a guanine base, a continuous irradiation of a solution containing conjugate **9** and GMP was achieved. The crude irradiation mixture was then analyzed by MALDI mass spectrometry (HRMS, Figure 6). Alongside the parent conjugate **9** ions, ionized species at higher mass-to-charge ratio (*m*/*z* = 4625.9) are detected and formally correspond to the addition of GMP minus two hydrogen atoms. The comparison between the experimental and theoretical isotope patterns confirms (inset Figure 6) that irradiation of conjugate **9** and GMP efficiently yielded the desired photoadduct.

## Conclusion

The present work validates the design strategy that consists in using calix[4]arenes as addressable platforms for the elaboration of multivalent photoreactive systems that could potentially target and enter cancer cells. The selective tethering of a photoreactive Ru-TAP complex on the small rim of a calix[4]arene and the introduction of four c-[RGDfK] moieties on its large rim were efficiently achieved. In good agreement with molecular modeling simulations, it was shown that the photophysical properties of the tethered complex **9** are not altered by the anchoring onto the calixarene platform and that the cyclic pentapeptide units do not interfere with the photoreactivity of the complex. Moreover, we verified that the complex is able to photoreact with its biological target, i.e., the guanine content of DNA, by demonstrating the occurrence of a photoinduced electron transfer and the formation of a covalent photoadduct between the Ru-calix(RGD)_4_ conjugate **9** and GMP. In conclusion, the ruthenium complex should be able to perform efficiently its photoinduced cytotoxic activity, once incorporated into targeted cancer cells thanks to the multivalent platform. In cellulo studies are currently under investigation and will be reported in the near future.

## Experimental

**General:** All the solvents and reagents for the syntheses were at least reagent grade quality and were used without further purification. Anhydrous *N*,*N*-dimethylformamide was purchased from ACROS Organics. Reactions were magnetically stirred and monitored by thin-layer chromatography using Fluka silica gel or aluminium oxide on TLC-PET foils with fluorescent indicator at 254 nm. All reactions involving ruthenium(II) were carried out in the dark. C18 reversed-phase silica gel (230−400 mesh) was used for chromatography. ^1^H NMR spectra were recorded at ambient temperature on Bruker 300, Variant 400 and 600 MHz spectrometers and ^13^C NMR spectra were recorded at 75, 100 or 150 MHz. Traces of residual solvents were used as internal standards for ^1^H NMR (7.26 ppm for CDCl_3_, 3.31 for CD_3_OD, 4.79 for D_2_O, 2.50 for DMSO-*d*_6_ and 1.94 ppm for CD_3_CN) and ^13^C NMR (77.16 ppm for CDCl_3_, 49.00 for CD_3_OD, 39.52 for DMSO-*d*_6_ and 118.26 ppm for CD_3_CN) chemical shift referencing. Abbreviations: s = singlet, d = doublet, t = triplet, q = quartet, br = broad, m = massif, mult = multiplet). 2D NMR spectra (COSY, HSQC, HMBC, HSQC) were recorded to complete signal assignments. Melting points were recorded on a Stuart Scientific Analogue SMP11 or Büchi Melting Point B-545. Infrared spectra were recorded on a Bruker Alpha (ATR) spectrometer.

High-resolution mass spectra were obtained on a Waters Synapt G2-Si spectrometer (Waters, Manchester, UK) equipped with an electrospray ionization used in the positive ion mode. Source parameters were as follow: capillary voltage, 3.1 kV; sampling cone, 30 V; source Offset, 80 V; source temperature, 150 °C and desolvation temperature, 200 °C. Matrix-assisted laser desorption/ionization time-of-flight (MALDI-ToF) mass spectra were recorded using a Waters QToF Premier mass spectrometer equipped with a Nd-YAG laser of 355 nm with a maximum pulse energy of 65 μJ delivered to the sample at 50 Hz repeating rate. Time-of-flight mass analyses were performed in the reflection mode at a resolution of about 10 000. The matrix, *trans*-2-(3-(4-*tert*-butylphenyl)-2-methyl-2-propenylidene)malononitrile, was prepared as a 40 mg/mL solution in chloroform. The matrix solution (1 μL) was applied to a stainless-steel target and air-dried. The crude photoirradiation product was dissolved in acetonitrile and 1 μL aliquot of this solution was applied onto the target area (already bearing the matrix crystals) and then air-dried.

The HPLC purification process on final compound **9** was performed on a semi-preparative Infinity Agilent 1290 UHPLC system equipped with a binary pump, a thermostatically controlled injection system, a thermostatically controlled column compartment and a Diode Array detector. Waters C18 (Atlantis T3) column was used and the elution conditions are described in Supporting Information File 1.

Calix[4]arenes **2** and **3** were synthesized from commercial *p*-*tert-*butylcalix[4]arene **1** according to procedures described in the literature [69]. The experimental procedures and characterization data for calixarene derivatives **4**, **6**, **7** and **9**, phenanthroline derivative **5** and c-[RGDfK]-alkyne **8** are given in Supporting Information File 1.

The UV–vis absorption spectra were recorded on a Perkin-Elmer Lambda UV–vis spectrophotometer and the emission spectra with a Shimadzu RF-5001 PC spectrometer (detection: Hamamatsu R-928 red-sensitive photomultiplier tube, excitation source: xenon lamp 250 W). Emission quantum yields were determined by integrating the corrected emission spectra over the frequencies. [Ru(bpy)_3_]^2+^ in water under air was chosen as the standard luminophore (quantum yield of 0.042 under argon). The luminescence lifetimes were measured by the time-correlated single photon counting (TC-SPC) technique with the Edinburgh Instruments LifeSpecII Picosecond Fluorescence Lifetime Spectrometer equipped with a laser diode (λ = 439 nm, pulse = 100 ps). The samples were thermostatted at 20 ± 2 °C with a Haake Model NB22 temperature controller. The data were collected by a multichannel analyzer (2048 channels) with a number of counts in the first channel equal to 104. The resulting decays were deconvoluted for the instrumental response and fitted to the exponential functions using the original manufacturer software package (Edinburgh Instruments). The reduced χ^2^, weighted residuals and autocorrelation function were employed to judge the quality of the fits.

For molecular modelling simulations, the initial structure of the ruthenium complex was obtained from previous DFT calculations using a previously reported methodology [82]. The geometries of the RAFTs and calixarene were built using DS BIOVIA^©^ software and geometry-optimized using the CHARMM force-field, taking into account previous molecular modelling simulations on cyclic peptides [83]. The structures were linked together yielding through several steps of energy minimizations, maintaining the ruthenium complex constrained in octahedral geometry by harmonic constraints. After energy minimization of the entire structure, MD simulations of 5 ns were produced in NVT ensemble at 300 K, in the generalized Born implicit solvent model. Although the MD simulation time used here is way insufficient to probe the conformational landscape of this large molecule, the conformations reported here represent relaxed geometries showing possible intermolecular contacts between the cyclic pentapeptides. The analysis and visualization of MD simulations were carried out using DS BIOVIA and Chimera [84] software.

## Supporting Information

File 1 Supplementary information.
