# Supplementary material for: Synthesis and photophysical studies of a multivalent photoreactive RuII-calix[4]arene complex bearing RGD-containing cyclopentapeptides
Source: Beilstein J Org Chem. 2018 Jul 16;14:1758–68. doi: 10.3762/bjoc.14.150 (PMC6071717; doi:10.3762/bjoc.14.150)
Supplement: File 1 — Supplementary information. [file Beilstein_J_Org_Chem-14-1758-s001.pdf]

Supporting Information

for

Synthesis and photophysical studies of a multivalent photoreactive Ru^{II}-calix[4]arene complex bearing RGD-containing cyclopentapeptides

Sofia Kajouj[1], Lionel Marcelis[*1,2], Alice Mattiuzzi[3], Adrien Grassin[4], Damien Dufour[5], Pierre Van Antwerpen[5], Didier Boturyn[4], Eric Defrancq[4], Mathieu Surin[6], Julien De Winter[7], Pascal Gerbaux[7], Ivan Jabin[*3], and Cécile Moucheron[*1]

Address: [1]Laboratoire de Chimie Organique et Photochimie, Université libre de Bruxelles, Avenue F.D. Roosevelt 50, CP 160/08, 1050 Bruxelles, Belgium, [2]Engineering of Molecular NanoSystems,Ecole Polytechnique de Bruxelles, Université libre de Bruxelles (ULB), Avenue F.D. Roosevelt 50, CP165/64, B-1050 Brussels, Belgium, [3]Laboratoire de Chimie Organique, Université libre de Bruxelles, Avenue F.D. Roosevelt 50, CP 160/06, 1050 Bruxelles, Belgium, [4]Université Grenoble Alpes, Département de Chimie Moléculaire UMR CNRS 5250, CS 40700, 38058 Grenoble Cedex 09, France, [5]Analytical Platform of the Faculty of Pharmacy, Université libre de Bruxelles, Boulevard du Triomphe, Campus de la Plaine, CP205/05, 1050 Bruxelles, Belgium, [6]Laboratory for Chemistry of Novel Materials, Center for Innovation and Research in Materials and Polymers, University of Mons – UMONS, 20, Place du Parc, B-7000 Mons, Belgium and [7]Organic synthesis and Mass Spectrometry Laboratory, University of Mons - UMONS, Place du Parc 23, B-7000 Mons, Belgium

Email: Lionel Marcelis - lmarceli@ulb.ac.be; Ivan Jabin - ijabin@ulb.ac.be; Cécile Moucheron - cmouche@ulb.ac.be

*Corresponding author

Supplementary Information

General information.	S2
Experimental Procedures and characterization data.	S4
Calixarene derivatives (**4**, **6**, **7** and **9**).	S4
Phenanthroline derivative **5**.	S14
c-[RGDfK]-alcyne **8**.	S15
Molecular modelling simulations of conjugate **9**.	S17
Quenching studies on conjugate **7**.	S18

General information.

All the solvents and reagents for the syntheses were at least reagent grade quality and were used without further purification. Anhydrous *N*,*N*-dimethylformamide was purchased from ACROS Organics. Reactions were magnetically stirred and monitored by thin layer chromatography using Fluka Silica gel or Aluminium oxide on TLC-PET foils with fluorescent indicator at 254 nm. All reactions involving ruthenium (II) were carried out in the dark. C18 reversed phase silica gel (230−400 mesh) was used for chromatography. ^{1}H NMR spectra were recorded at ambient temperature on Bruker 300, Variant 400 and 600 MHz spectrometers and ^{13}C NMR spectra were recorded at 75, 100 or 150 MHz. Traces of residual solvents were used as internal standards for ^{1}H (7.26 ppm for $CDCl_3$, 3.31 for CD_3OD, 4.79 for D_2O, 2.50 for DMSO-*d*6 and 1.94 ppm for CD_3CN) and ^{13}C (77.16 ppm for $CDCl_3$, 49.00 for CD_3OD, 39.52 for DMSO-*d*6 and 118.26 ppm for CD_3CN) chemical shift referencing. Abbreviations: s = singlet, d = doublet, t = triplet, q = quartet, br = broad, m = massif, mult = multiplet). 2D NMR spectra (COSY, HSQC, HMBC, HSQC) were recorded to complete signal assignments. Melting points were recorded on a Stuart Scientific Analogue SMP11 or Büchi Melting Point B-545. Infrared spectra were recorded on a Bruker Alpha (ATR) spectrometer. High-resolution mass-spectra were obtained on a Synapt G2-Si spectrometer (Waters, Manchester, UK) equipped with a Quadrupole Time of Flight (QTOF) using the following settings: capillary voltage, 3.1 kV; sampling cone, 30 V; source Offset, 80 V; source temperature, 150°C and desolvation temperature, 200 °C. HPLC purification process on final compound **9** was performed on a semi-preparative Infinity Agilent 1290 UHPLC equipped with a binary pump, a thermostatically controlled injection system, a thermostatically controlled column compartment and a Diode Array detector. Waters C18 (Atlantis T3) column was used and the elution conditions are described in the following table.

**mL/min**	Time **(min)**	**H_2O/TFA(0.1%)**	**ACN/TFA(0.1%)**
**5**	0	90	10
**5**	1	90	10
**5**	40	60	40
**5**	50	5	95
**5**	60	5	95
**5**	61	90	10
**5**	80	90	10

The starting calixarene *p*-*t*Bu-calix[4]arene **1** and Fmoc protected amino-acids are commercial; other starting materials such as calixarenes **2** and **3**,[1] *N*-hydrosuccinimide ester of (*N*-Boc-amino)acetate and 5-glycinamido-1,10-phenanthroline, [2] and alkyne-modified Lysine[3] were synthesized according to procedures described in the literature.

---

[1] Mattiuzzi, A.; Jabin, I.; Mangeney, C.; Roux, C.; Reinaud, O.; Santos, L.; Bergamini, J.-F.; Hapiot, P.; Lagrost, C. *Nat. Comm.*, **2012**, *3*, 1-8.

[2] Deroo, S.; Defrancq, E.; Moucheron, C.; Kirsch-De Mesmaeker, A.; Dumy, P. *Tetrahedron Lett.*, **2003**, *44*, 8379-8382.

[3] Galibert, M.; Sancey, L.; Renaudet, O.; Coll, J.-L.; Dumy, P.; Boturyn, D. *Org. Biomol. Chem.*, **2010**, 8, 5133-5138.

Experimental Procedures and characterization data.
Calixarene derivatives (4, 6, 7 and 9).

Calix[4]arene 4. $NaNO_2$ (225 mg, 3.26 mmol) was added in a mixture containing the tetra-amino **3** (500 mg, 0.73 mmol) in 45 mL of 10% aqueous HCl cooled at 0°C. The mixture was stirred at 0 °C for 30 min. A solution of sodium azide (282 mg, 4.34 mmol) in 2 mL of water was then added dropwise. A vigorous gas evolution was observed. The solution was allowed to warm up to room temperature. After 5 hours, the reaction was stopped by slowly adding a NaOH solution (11.7 g, 292.4 mmol in 20 mL water). The aqueous layer was extracted twice with CH_2Cl_2 (3 x 100 mL) and the organic layer was washed twice with water (2 x 100 mL). The organic solution was dried over Na_2SO_4, filtered and CH_2Cl_2 was evaporated, providing the compound **4** as a light brown solid (329 mg, 56%). Mp: 159 °C. ^1H NMR (400 MHz, $CDCl_3$, 298 K) δ (ppm): 6.83 (4H, s, ArH), 5.94 (2H, d, ^4J = 2.7 Hz, ArH), 5.89 (2H, d, ^4J = 2.7 Hz, ArH), 4.80 (2H, s, OCH_2CO), 4.41 (2H, d, ^2J = 13.5 Hz, $ArCH_2^{ax}$), 4.3 (2H, d, ^2J = 14.1 Hz, $ArCH_2^{ax}$), 3.90 (2H, t, ^3J = 8.2 Hz, $OCH_2CH_2CH_3$), 3.73 (4H, t, ^3J = 7.3 Hz, $OCH_2CH_2CH_3$), 3.26 (2H, d, ^2J = 14.1 Hz, $ArCH_2^{eq}$), 3.14 (2H, d, ^2J = 13.5 Hz, $ArCH_2^{eq}$), 1.91-1.77 (6H, m, $CH_2CH_2CH_3$), 1.02 (6H, t, ^3J = 7.5 Hz, $CH_2CH_2CH_3$), 0.84 (3H, t, ^3J = 7.6 Hz. $CH_2CH_2CH_3$). ^{13}C NMR (75 MHz, $CDCl_3$, 298 K) δ (ppm): 170.4, 154.5, 154.0, 151.2, 137.8, 136.2, 135.6, 134.9(7), 134.9(5), 134.5, 133.6, 120.1, 119.5, 118.8, 118.2, 78.7, 71.9, 31.6, 31.2, 23.0, 22.7, 10.5, 9.8. IR (ATR) υ_{max}: 2108, 1468, 1233, 1206, 1114, 963, 856 cm^{-1}. HRMS (ESI+): *m/z* calculated for $C_{39}H_{40}N_{12}O_6Na$ $[M+Na]^+$ 795.3091, found 795.3080.

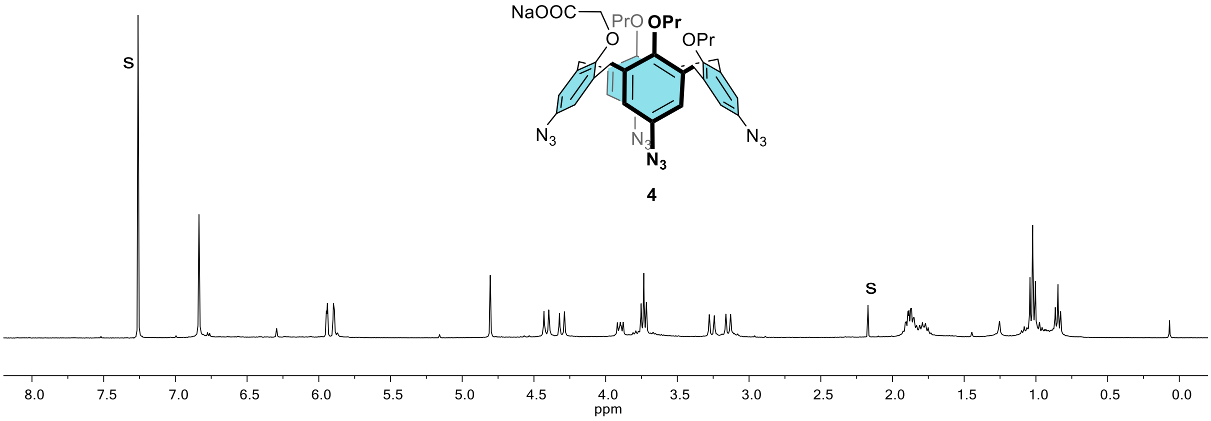

Figure S1: ^1H NMR spectrum (400MHz, 298K) of calix[4]arene **4** in $CDCl_3$. s: residual solvents.

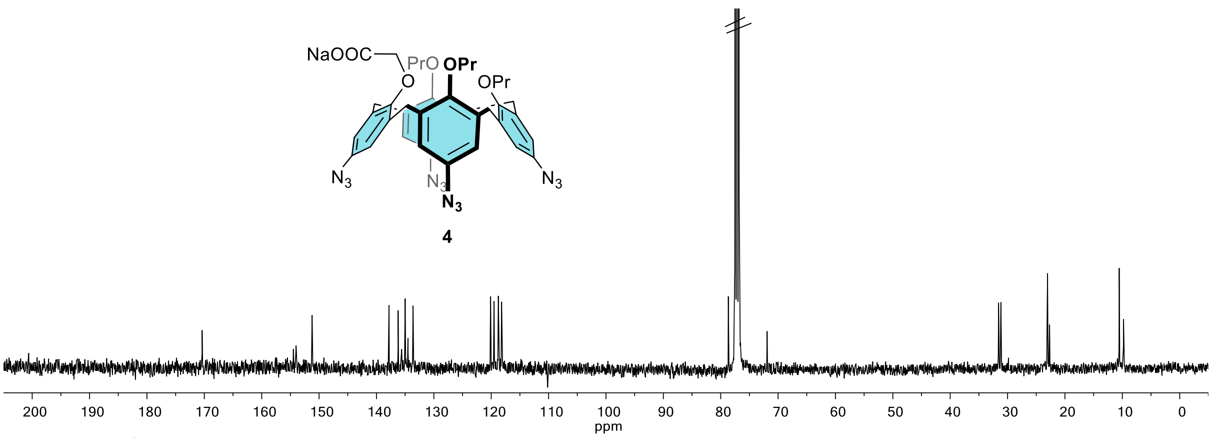

Figure S2: ^{13}C NMR spectrum (75MHz, 298K) of calix[4]arene **4** in $CDCl_3$.

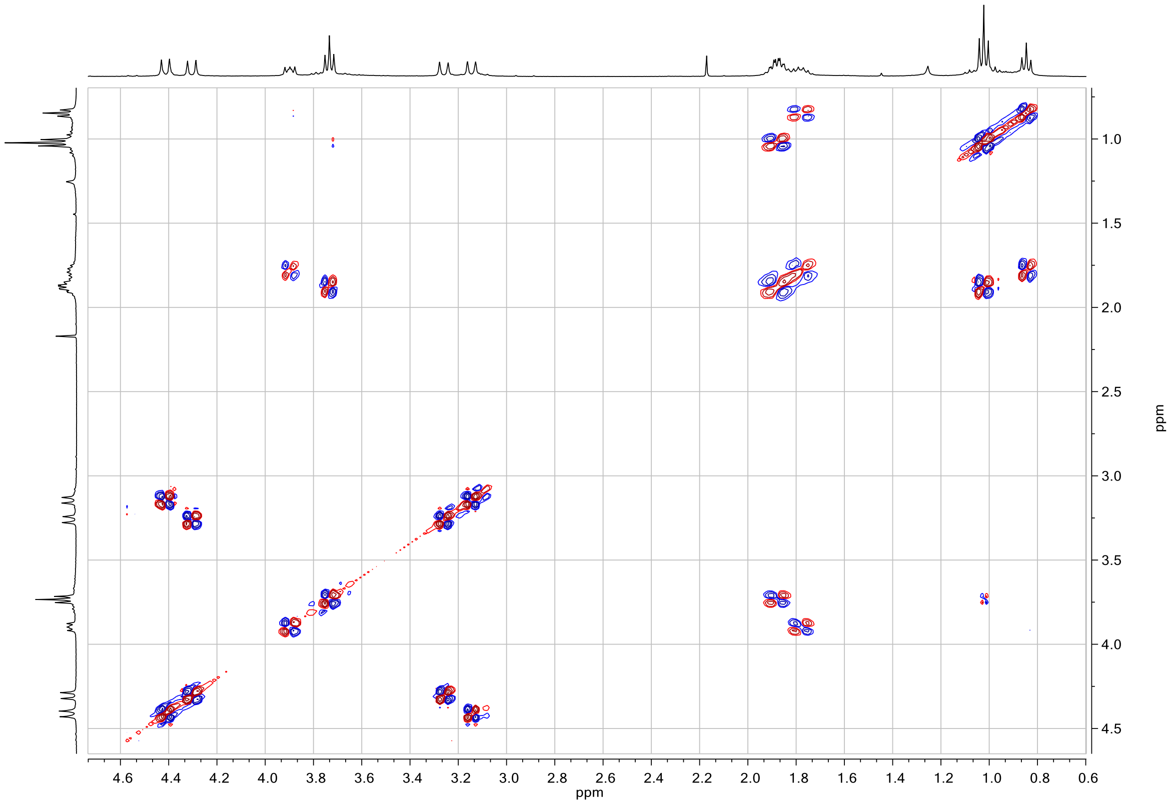

Figure S3: COSY spectrum (400MHz, 298K) of calix[4]arene **4** in $CDCl_3$.

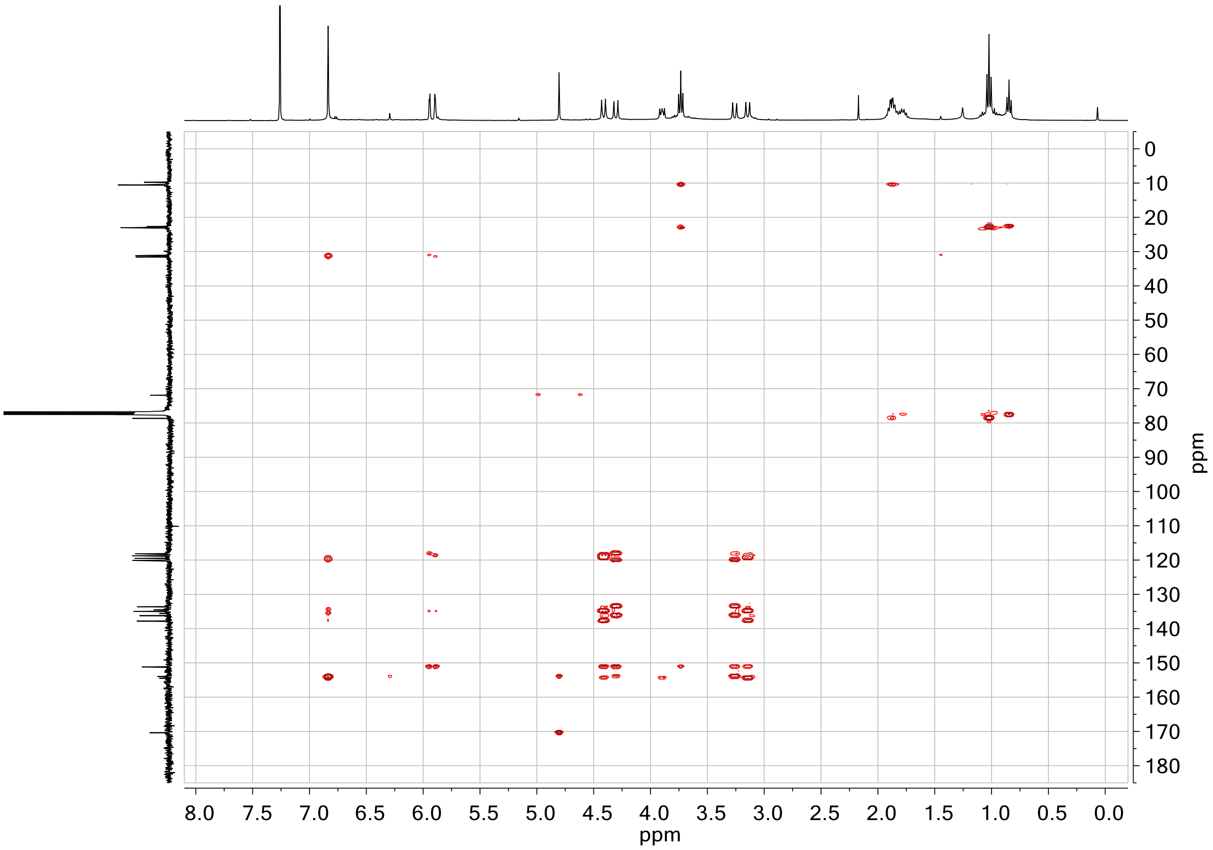

Figure S4: HMBC spectrum (400MHz, 298K) of calix[4]arene **4** in $CDCl_3$.

Calix[4]arene 6. Calix[4]arene **4** (116 mg, 0.15 mmol), EDC.HCl (57.5 mg, 0.3 mmol) and HOBt (40.5 mg, 0.3 mmol) were dissolved in DMF (1.5 mL) and the solution was cooled at 0°C. After addition of DIPEA (300 µL, 1.8 mmol), the mixture was stirred for 30 min at 0°C under inert atmosphere. The freshly prepared compound **5** (127 mg, 0.3 mmol), previously dissolved in DMF (1.5 mL), was added to the mixture. After two days of stirring at room temperature, an excess of water was added to the reaction mixture to induce precipitation of the product. The resulting precipitate was washed two times with an excess of a 5% HCl solution and two times with an excess of water, leading to target compound **6** as a beige solid (150 mg, 0.14 mmol, 94%). Mp: 190 °C (dec.). ^{1}H NMR (400 MHz, DMSO-d_6, 298 K) δ (ppm): 10.23 (1H, s, NH$_\alpha$), 9.15 (1H, dd, ^3J = 3.6 Hz, ^4J = 1.6 Hz, H$_2$), 9.07 (1H, dd, ^3J = 3.6 Hz, ^4J = 1.6 Hz, H$_9$), 8.69 (1H, d, ^3J = 8.8 Hz, H$_4$), 8.60-8.55 (2H, m, H$_7$ + H$_\varepsilon$), 8.34 (1H, t, ^3J = 6.0 Hz, H$_\gamma$), 8.19 (1H, s, H$_6$), 7.87-7.82 (2H, m, H$_3$ + H$_8$), 6.46 (4H, s, ArH), 6.34-6.32 (4H, m, ArH), 4.50 (2H, s, OC*H*$_2$CO), 4.41 (2H, d, ^2J = 14.0 Hz, ArCH$_2^{ax}$), 4.25 (2H, d, ^2J = 13.2 Hz, ArCH$_2^{ax}$), 4.16 (2H, d, ^3J = 5.6 Hz, H$_\delta$), 4.02 (2H, d, ^3J = 5.6 Hz, H$_\beta$), 3.85-3.69 (6H, m, OC*H*$_2$CH$_2$CH$_3$), 3.11-3.24 (4H, m, ArCH$_2^{eq}$), 1.83-1.70 (6H, m, CH$_2$C*H*$_2$CH$_3$), 0.92-0.90 (3H, t, ^3J = 7.6 Hz, CH$_2$CH$_2$C*H*$_3$), 0.86 (6H, t, ^3J = 7.2 Hz, CH$_2$CH$_2$C*H*$_3$).^{13}C NMR (75 MHz, DMSO-d_6, 298 K) δ (ppm): 169.9, 169.5, 169.3, 154.7, 154.5, 154.1, 153.9, 150.3, 149.2, 136.9, 136.7, 136.3, 136.2, 133.5, 133.2(4), 133.2(1), 133.1, 132.4, 128.8, 125.5, 124.5, 123.8, 119.3, 119.1, 119.0, 118.9, 77.3, 76.8, 30.9, 30.6, 23.1, 22.9 (2C), 10.6 (2C). IR υ_{max}: 2867, 2112, 1683, 1467, 1234, 1053, 1000 cm^{-1}. HRMS (ESI+): *m/z* calculated for $C_{55}H_{53}N_{17}O_7Na$ [M+Na]$^+$ 1086.4212, found 1086.4194.

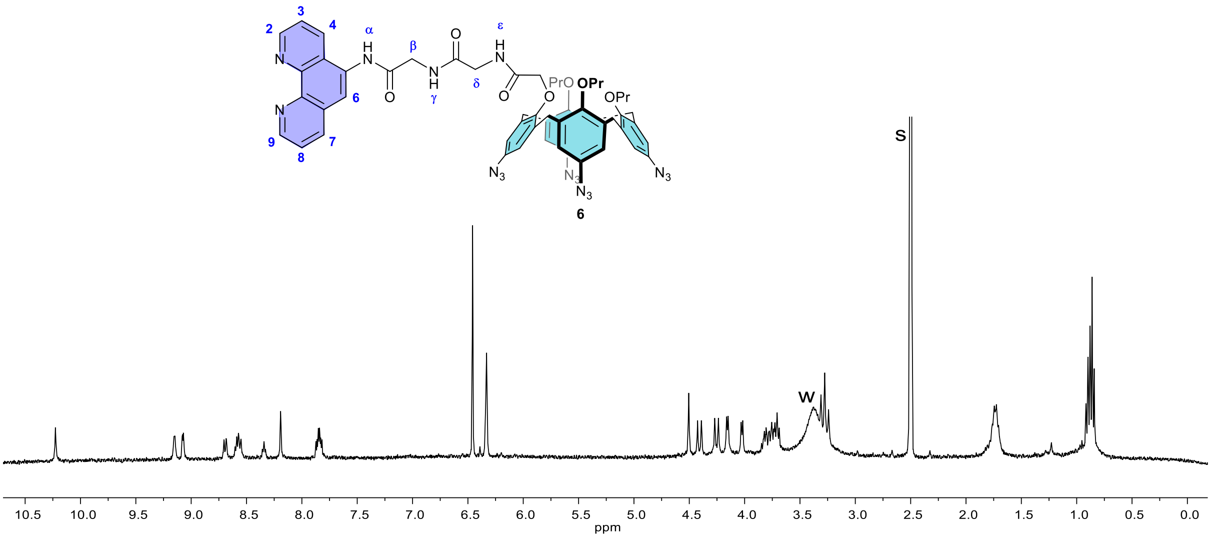

Figure S5: ^{1}H NMR spectrum (400 MHz, 298K) of calix[4]arene **6** in DMSO-d6. s: residual solvent. w: water.

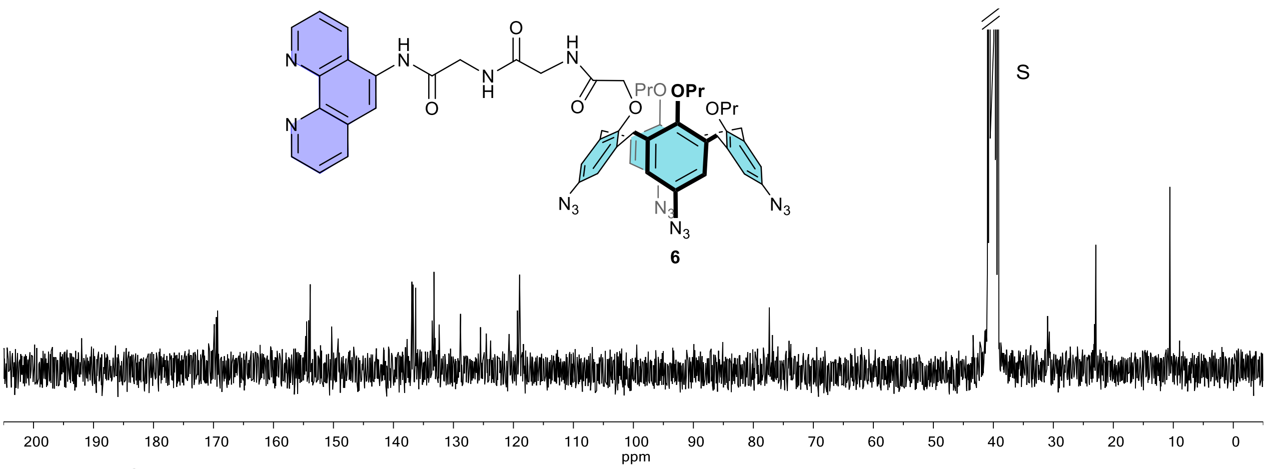

Figure S6: ^{13}C NMR spectrum (75 MHz, 298K) of calix[4]arene **6** in DMSO-d6.

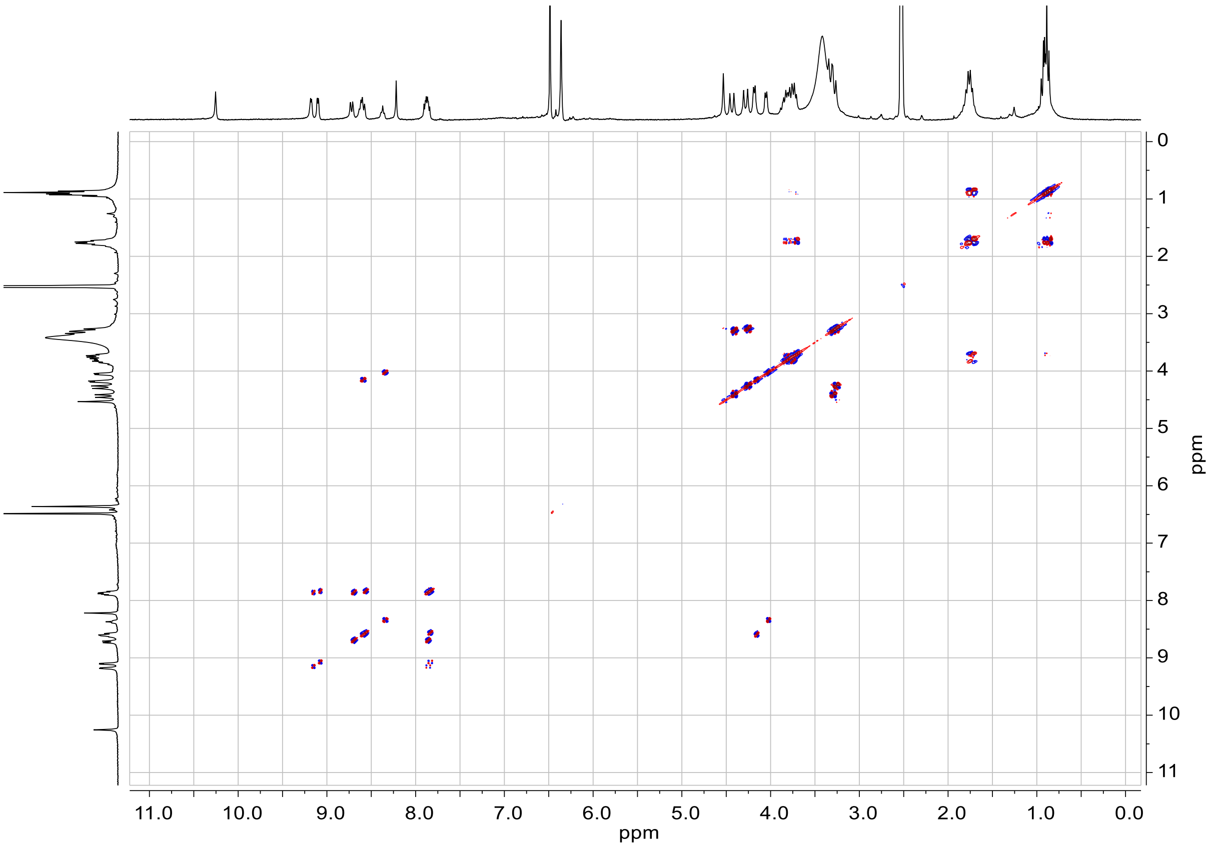

Figure S7: COSY spectrum (400 MHz, 298K) of calix[4]arene **6** in DMSO-d6.

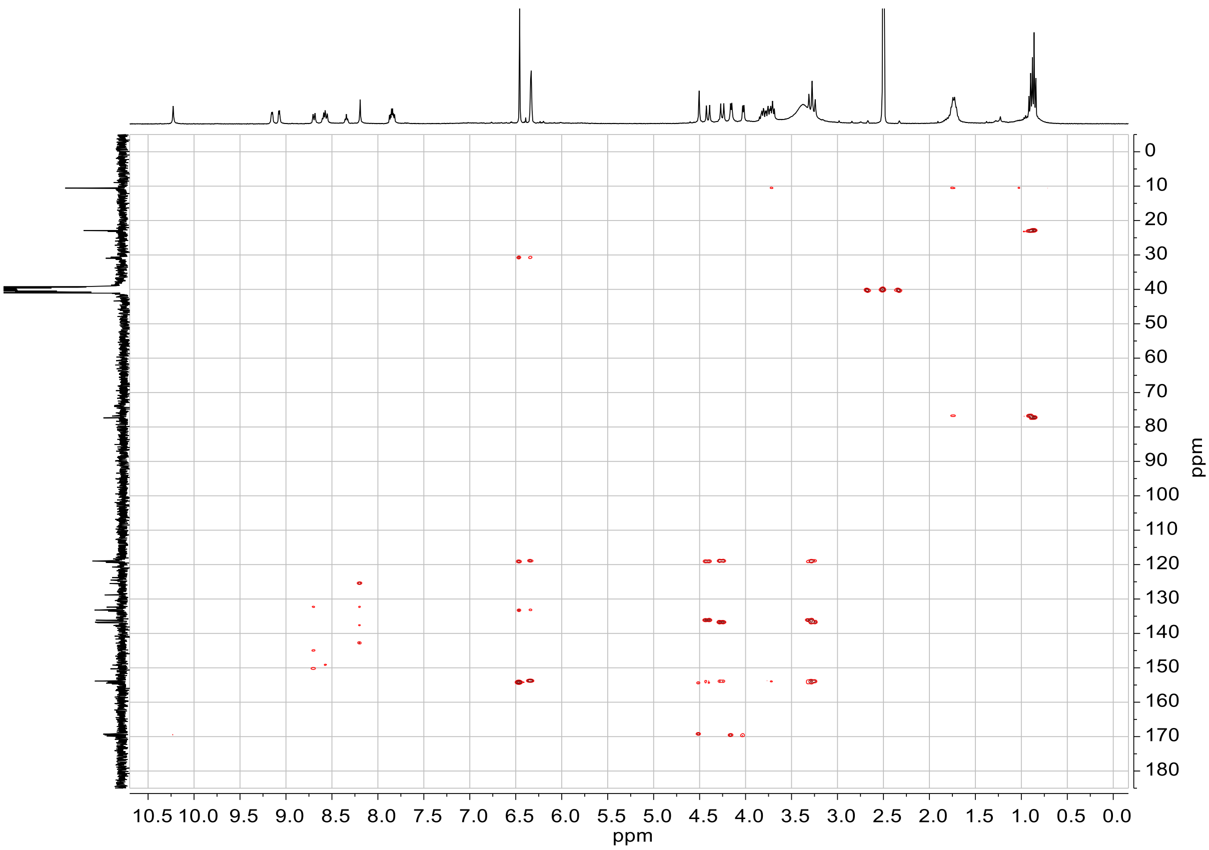

Figure S85: HMBC spectrum (400 MHz, 298K) of calix[4]arene **6** in DMSO-d_6.

Ru^{II}-calix[4]arene complex 7. Calix[4]arene **6** (40 mg, 0.038 mmol) was dissolved in DMF (5 mL) and Ru(TAP)$_2$(H_2O)$_2$ (22.6 mg, 0.045 mmol) was added. The reaction mixture was stirred for 3h at 100°C under inert atmosphere to yield a dark-red solution. The reaction mixture was concentrated under reduced pressure and the crude residue was purified by C18 silica gel chromatography (CH_3CN/H_2O; 9:1) to yield the Ru^{II}-calix[4]arene complex **7** as a dark brown solid (64 mg, 0.036 mmol, 95%). ^{1}H NMR (600 MHz, CD_3CN, 298 K) δ (ppm): 10.33 (1H, s, NH$_\alpha$), 9.00 (1H, d, ^3J = 8.4 Hz, H$^P{}_4$), 8.96-8.92 (4H, m, H$^T{}_{2,2',7,7'}$), 8.62-8.56 (6H, m, H$^P{}_6$ + H$^P{}_7$ + H$^T{}_{9,9',10,10'}$), 8.53 (1H, t, ^3J = 5.4 Hz, H$_\gamma$), 8.30 (1H, t, ^3J = 5.4 Hz, H$_\varepsilon$), 8.25 (1H, d, ^3J = 3.0 Hz, H$^T{}_6$), 8.23 (1H, d, ^3J = 3.0 Hz, H$^T{}_{6'}$), 8.16 (1H, d, ^3J = 3.0 Hz, H$^T{}_3$), 8.14 (1H, d, ^3J = 3.0 Hz, H$^T{}_{3'}$), 8.10 (1H, dd, ^3J = 5.4 Hz, ^{4}J = 1.2 Hz, H$^P{}_2$), 8.02 (1H, dd, ^3J = 5.4 Hz, ^4J = 1.2 Hz, H$^P{}_9$), 7.65-7.62 (2H, m, H$^P{}_3$ + H$^P{}_8$), 6.76-6.74 (2H, m, ArH), 6.68 (1H, d, ^3J = 3.0 Hz, ArH), 6.63 (1H, d, ^3J = 3.0 Hz, ArH), 6.06-6.02 (4H, m, ArH) 4.69 (2H, s, OCH_2CO), 4.43 (1H, d, ^2J = 13.8 Hz, ArCH$_2^{ax}$), 4.42 (1H, d, ^2J = 14.4 Hz, ArCH$_2^{ax}$), 4.35 (2H, d, ^2J = 13.8 Hz, ArCH$_2^{ax}$), 4.20 (2H, d, ^3J = 5.4 Hz, H$_\delta$), 4.13 (2H, d, ^3J = 6.0 Hz, H$_\beta$), 3.81 (2H, t, ^3J = 7.2 Hz, OCH_2CH$_2$CH$_3$), 3.77 (4H, t, ^3J = 7.2 Hz, OCH_2CH$_2$CH$_3$), 3.25-3.19 (4H, m, ArCH$_2^{eq}$), 1.77-1.69 (6H, m, CH$_2$CH_2CH$_3$), 0.86 (6H, m, CH$_2$CH$_2$CH_3), 0.83 (3H, t, ^3J = 7.2 Hz, CH$_2$CH$_2$CH_3). Given the complex C_1 structure of compound **7**, we were not able to obtain a ^{13}C NMR spectrum displaying signals of sufficient intensity even after 14000 scans (*ca.* 15 h of NMR measurement). HRMS (ESI+): *m/z* calculated for $C_{75}H_{65}N_{25}O_7Ru^{2+}$ [M]$^{2+}$ 764.7264, found 764.7281.

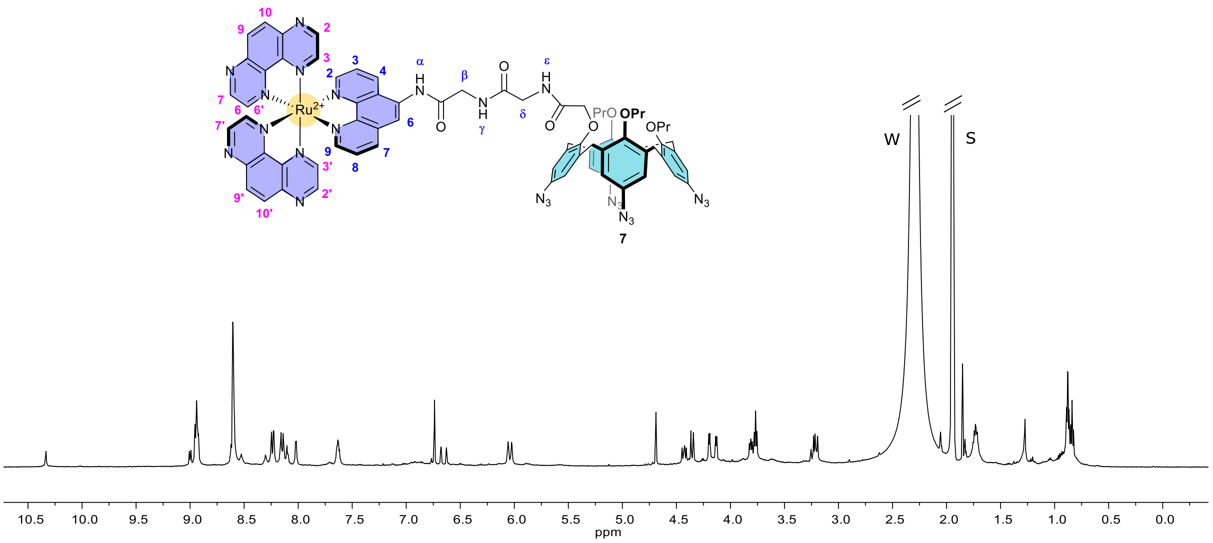

Figure S9: ^{1}H NMR spectrum (600 MHz, 298K) of Ru^{II}-calix[4]arene complex **7** in CD_3CN. s: residual solvent, w: residual water.

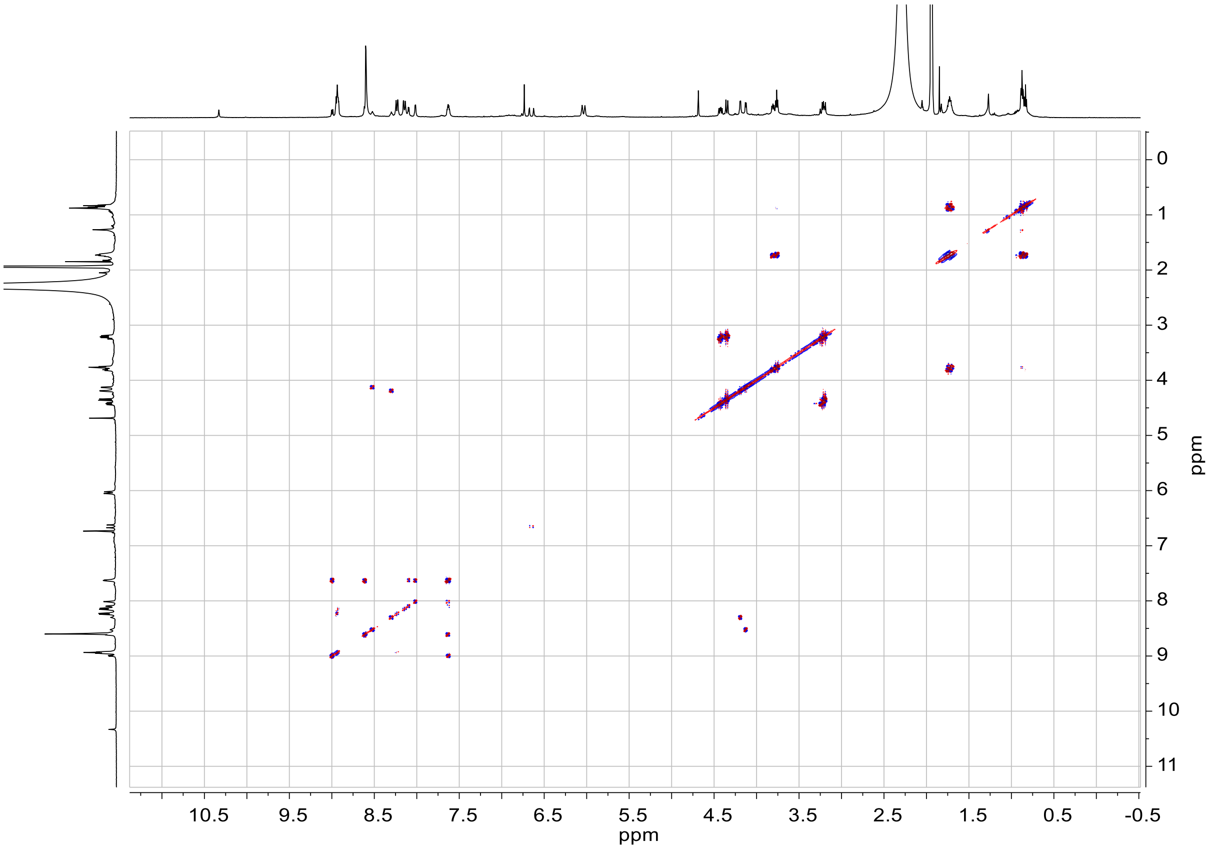

Figure S60: COSY spectrum (600 MHz, 298K) of Ru^{II}-calix[4]arene complex **7** in CD_3CN.

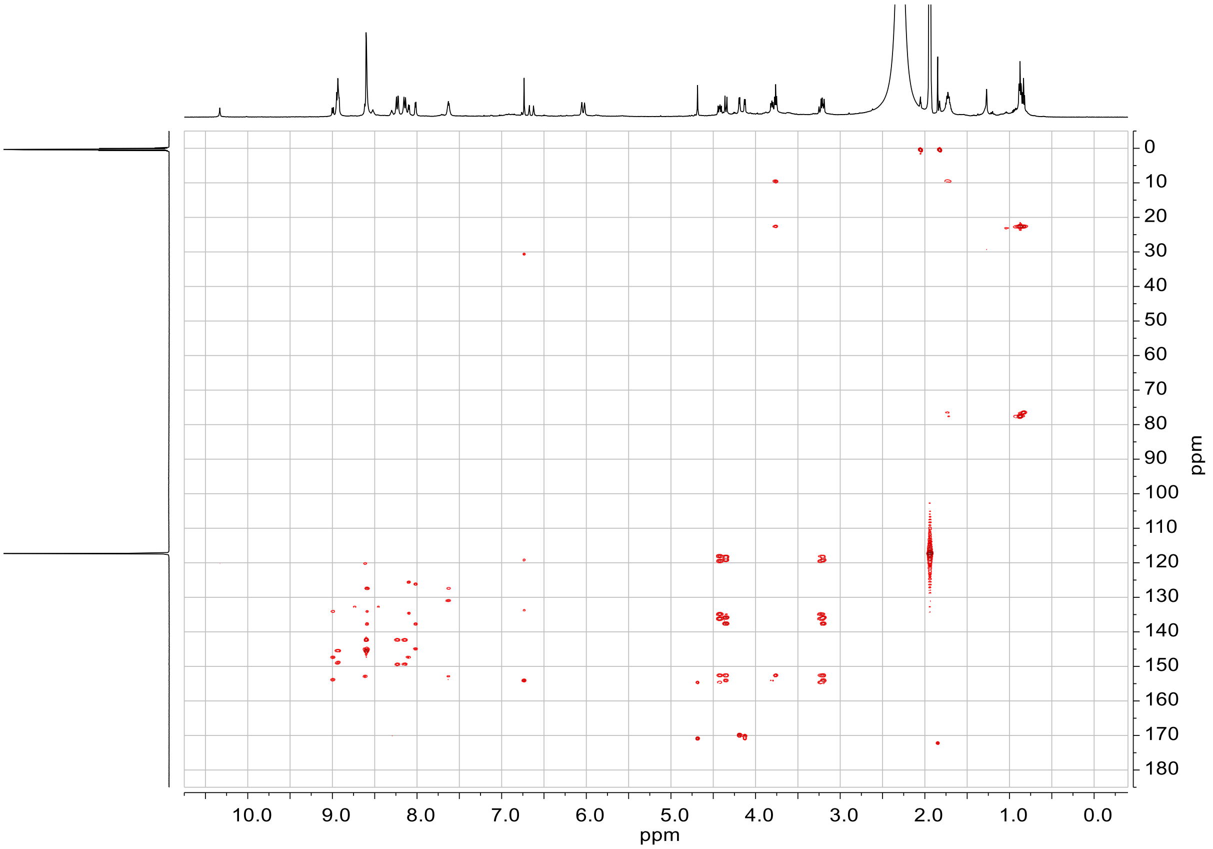

Figure S11: HMBC spectrum (600 MHz, 298K) of Ru^{II}-calix[4]arene complex **7** in CD_3CN.

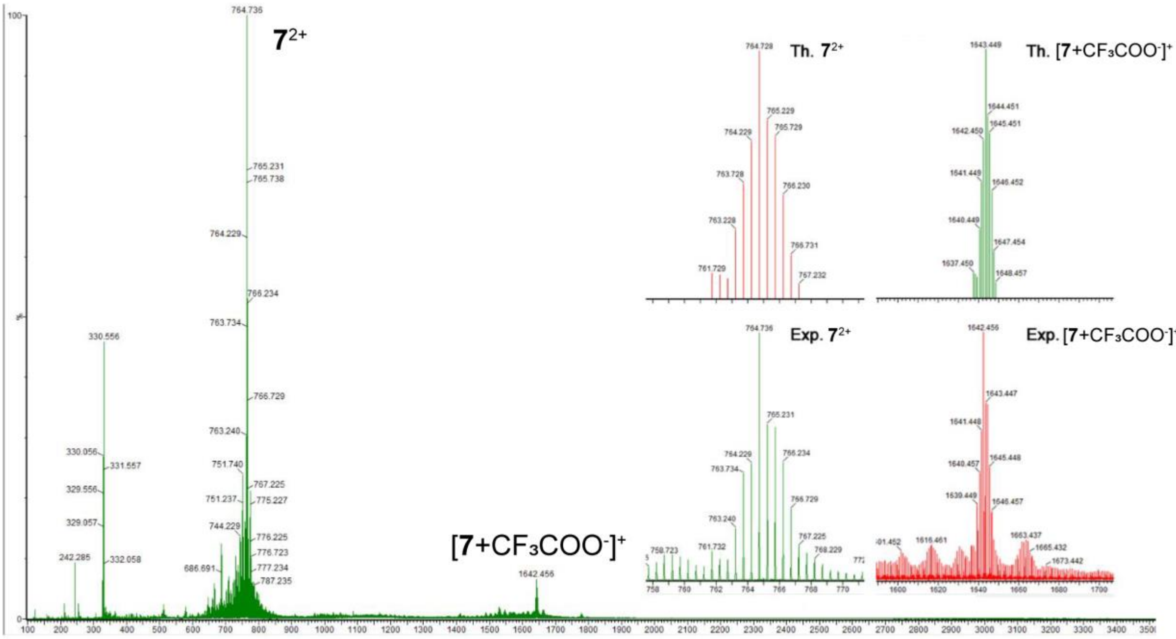

Figure S12: HRMS (ESI+) analysis conjugate **7**. In inset, the experimental and theoretical isotope distributions are compared for both $\mathbf{7}^{2+}$ and $[\mathbf{7}+CF_3COO^-]^+$ ions.

Ru^{II}-calix[4]arene-[c-(RGDfK)]$_4$ conjugate 9. Ru^{II}-calix[4]arene complex **7** (5 mg, 3 µmol) and c-(RGDfK)-alcyne **8** (9.7 mg, 15 µmol) were dissolved in DMF (0.5 mL) and copper nanoparticles (1.5 mg) were added. The synthesis was performed using micro-wave heating according to the following parameters: temperature = 50°C, run time = 30 sec, hold time = 60 min, medium stirring and power = 100W. The reaction mixture was concentrated under reduced pressure and the crude residue was purified by semi-preparative HPLC using an Atlantis T3 column (CH_3CN/H_2O/TFA; see the "General information" section for the details). The Ru^{II}-calix[4]arene-[c-(RGDfK)]$_4$ conjugate **9** was obtained as a brown solid (3.9 mg, 0.9 µmol, 31%). HRMS (ESI+): *m/z* calculated for $C_{203}H_{248}N_{61}O_{39}Ru^{2+}$ $[M+3H]^{5+}$ 853.568, found 853.556, *m/z* calculated for $C_{203}H_{247}N_{61}O_{39}Ru^{2+}$ $[M+2H]^{4+}$ 1066.205, found 1066.434, *m/z* calculated for $C_{203}H_{246}N_{61}O_{39}Ru^{2+}$ $[M+H]^{3+}$ 1421.606, found 1421.577.

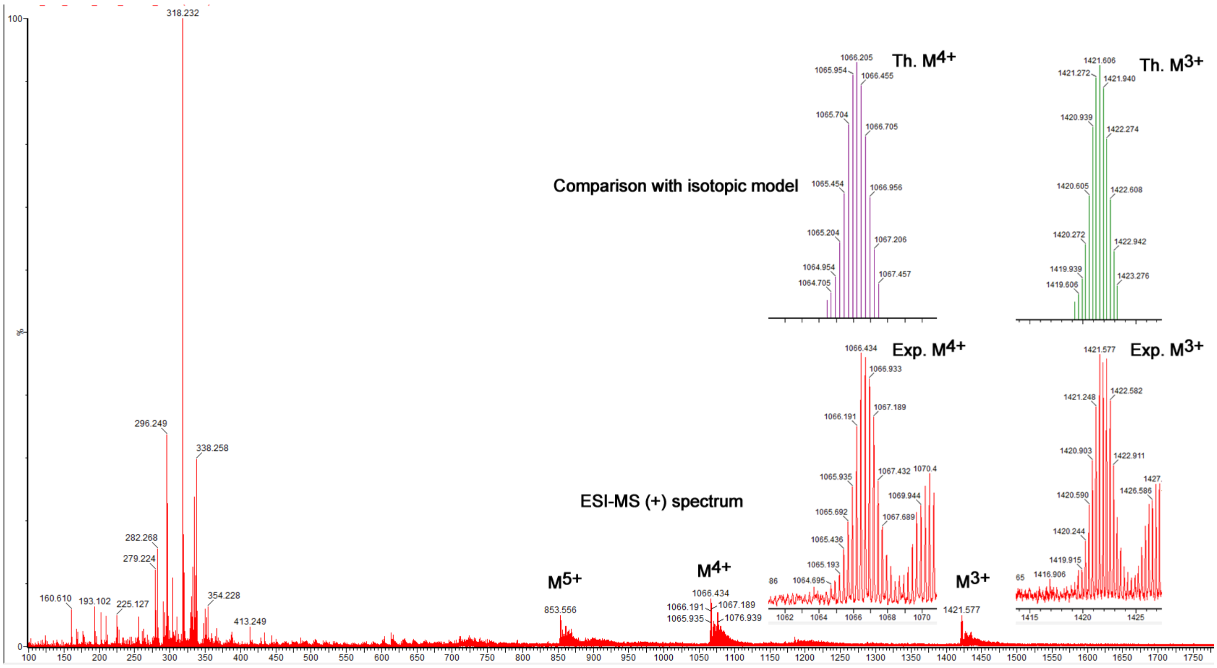

Figure S13: HRMS (ESI+) spectrum of Ru^{II}-calix[4]arene-[c-(RGDfK)]$_4$ conjugate **9**; [**9**+H]$^{3+}$, [**9**+2H]$^{4+}$ and [**9**+3H]$^{5+}$

Phenanthroline derivative 5.

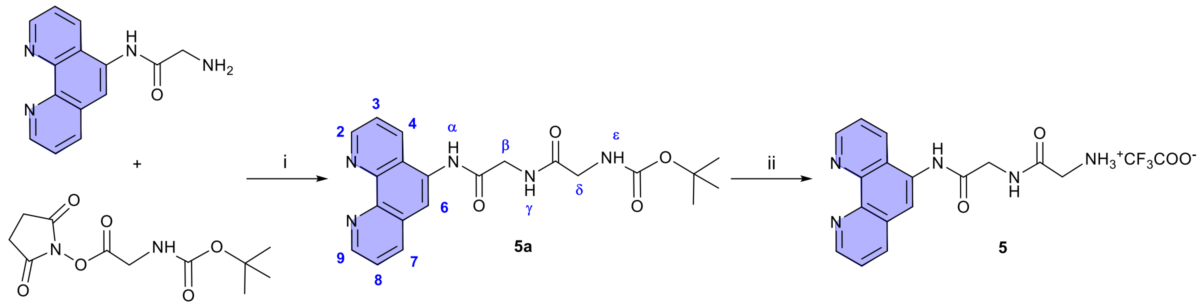

i) *N*-hydrosuccinimide ester of (*N*-Boc-amino)acetate (622 mg, 2.3 mmol) and 5-glycinamido-1,10-phenanthroline (607 mg, 2.4 mmol) were dissolved in DMF (20 mL) at room temperature under argon. The pH mixture was adjusted to 10 by addition of *N*,*N*-diisopropylethylamine (DIPEA). Stirring was maintained at room temperature for 1 hour under argon. The solvent was then removed under reduced pressure and the product was purified by chromatography on neutral alumina (eluent CH_2Cl_2/MeOH 98:2 to 95:5). The compound **5a** was obtained as a white solid (452 mg, 48%). ^{1}H NMR (300 MHz, CD_3OD, 298 K) δ (ppm): 9.13 (1H, dd, ^3J = 4.5 Hz, ^4J = 1.8 Hz, H_2), 9.08 (1H, dd, ^3J = 4.5 Hz, ^4J = 1.8 Hz, H_9), 8.62 (1H, dd, ^3J = 8.4 Hz, ^4J = 1.5 Hz, H_4), 8.42 (1H, dd, ^3J = 8.4 Hz, ^4J =1.8 Hz, H_7), 8.07 (1H, s, H_6), 7.82 (1H, dd, ^3J = 8.4 Hz, ^3J = 4.2 Hz, H_3), 7.77 (1H, dd, ^3J = 8.1 Hz, ^3J = 4.2 Hz, H_8), 4.24 (2H, s, H_δ), 3.84 (2H, d, ^3J = 6.0 Hz, H_β), 1.37 (9H, s, *t*Bu). ^{13}C NMR (75 MHz, CD_3OD, 298 K) δ (ppm): 173.7, 171.6, 151.0, 150.8, 147.1, 145.5, 137.7, 133.6, 132.6, 129.7, 126.9, 125.0, 124.5, 123.5, 84.2, 80.9, 45.1, 44.2, 34.8, 28.6. IR υ_{max}: 1673, 1513, 1367, 1248, 1168, 740 cm^{-1}. ESI-MS: *m/z* calculated for $C_{21}H_{24}N_5O_4$ $[M+H]^+$ 410.2, found 410.07 ; *m/z* calculated for $C_{42}H_{46}N_{10}O_8Na$ $[2M+Na]^+$ 841.4, found 841.0.

ii) Compound **5a** (100 mg, 0.24 mmol) was stirred in a 1:1 CH_2Cl_2/TFA mixture (25 mL) at room temperature for 90 min. The solvent was then removed under reduced pressure and the resulting product was washed several times with water and diethyl ether to eliminate the residual TFA. Compound **5** was obtained in quantitative yield as an orange oil and was directly engaged in the peptide type coupling reaction without any further purification. ^{1}H NMR (300 MHz, D_2O, 298 K) δ (ppm): 8.74 (1H, d, ^3J = 3.9 Hz, H_2), 8.66 (1H, d, ^3J = 4.8 Hz, H_9), 8.43 (1H, d, ^3J = 8.1 Hz, H_4), 8.35 (1H, d, ^3J = 8.4 Hz, H_7),7.75 (1H, s, H_6), 7.66 (2H, m, H_3 + H_8), 4.26 (2H, s, H_γ), 3.92 (2H, s, H_β).

c-[RGDfK]-alcyne 8.

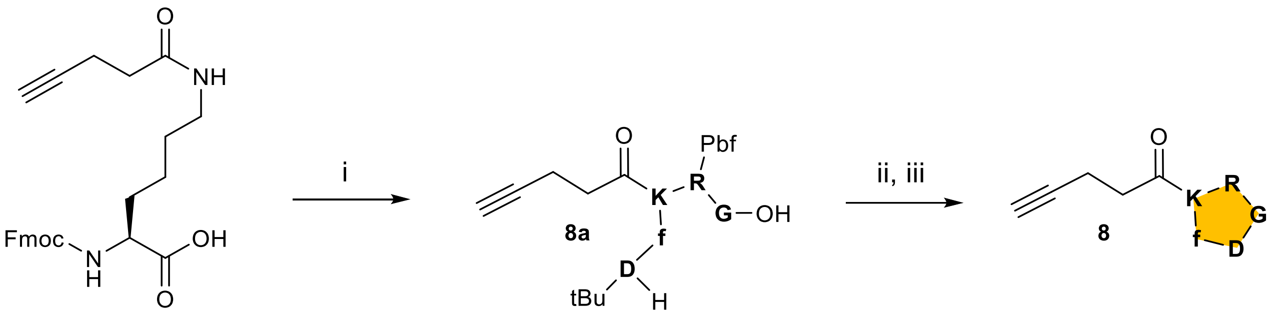

i) Analogous as described in the literature [1], the linear pentapeptide H-Asp(tBu)-D-Phe-Lys(*N*-4-Pentynoic acid)-Arg(Pbf)-Gly-OH **8a** was assembled according to the general procedure for solid phase peptide synthesis (500 mg, loading of 0.52 mmol/g). Coupling reactions were performed manually by using 2 equiv. of *N*-Fmoc-protected amino acid (relative to the resin loading) activated in situ with 2 equiv. of PyBOP and 3-5 equiv. of diisopropylethylamine (DIPEA) in DMF (10 mL/g resin) for 30 min. The coupling efficiency in manual synthesis was assessed by TNBS tests. *N*-Fmoc protecting groups were removed by treatment with a piperidine/DMF solution (1:4) for 10 min (10 mL/g resin). The process was repeated three times and the deprotection was verified by reading the absorbance of the piperidine washings at 299 nm. The linear peptide **8a** was then released from the resin by treatments with a solution of trifluoroacetic acid/methylene chloride (1:99, 10 mL/mg resin, 2x30 min). After evaporation, diethyl ether was added to the resulting crude peptide **8a** that was then triturated and washed three times with diethyl ether.

ii) Similarly to the previously reported method [1], the linear peptide **8a** was then dissolved in DMF (0.5 mM) and the pH values were adjusted to 8-9 by addition of DIPEA. PyBOP (1.3 equiv.) was added and the solution was stirred at room temperature for 1 h. Solvent was removed under reduced pressure and the residue was dissolved in a minimum of DCM. Diethyl ether was added to precipitate the cyclopeptide that was then triturated and washed three times with diethyl ether. The protected cyclopeptide was used in the next step without further purification.

iii) The protecting groups of the cyclopeptide were removed using a TFA/H_2O/TIS (95:2.5:2.5) solution. After 1 h of reaction, the reaction mixture was concentrated under vacuum providing an oily residue. Pure compound **8** was obtained as a white powder after purification using preparative RP-HPLC (117 mg, 0.17 mmol, 66% yield over 3 steps).

MS (ESI+): *m/z* calculated for $C_{32}H_{45}N_9O_8$: 683.8, found 683.5.

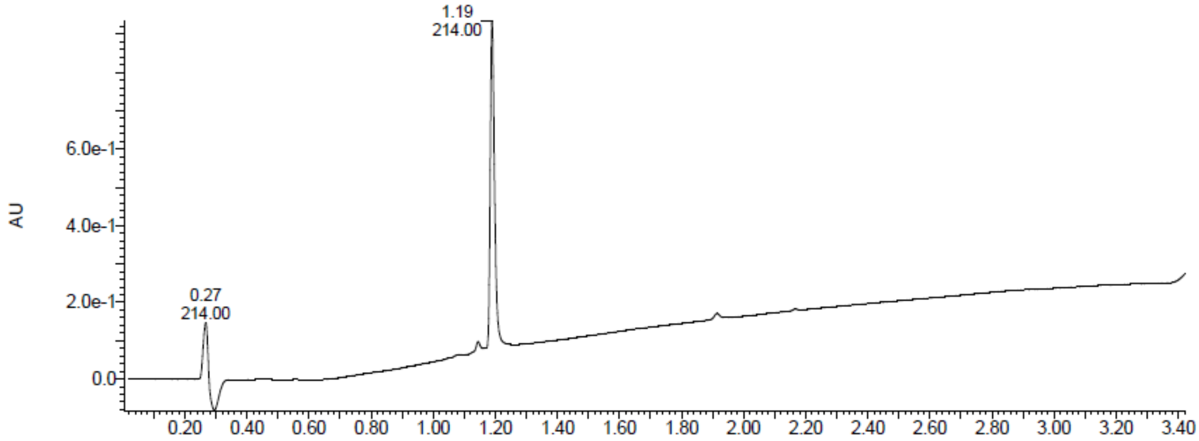

Figure S14: UPLC analysis of compound **8**.

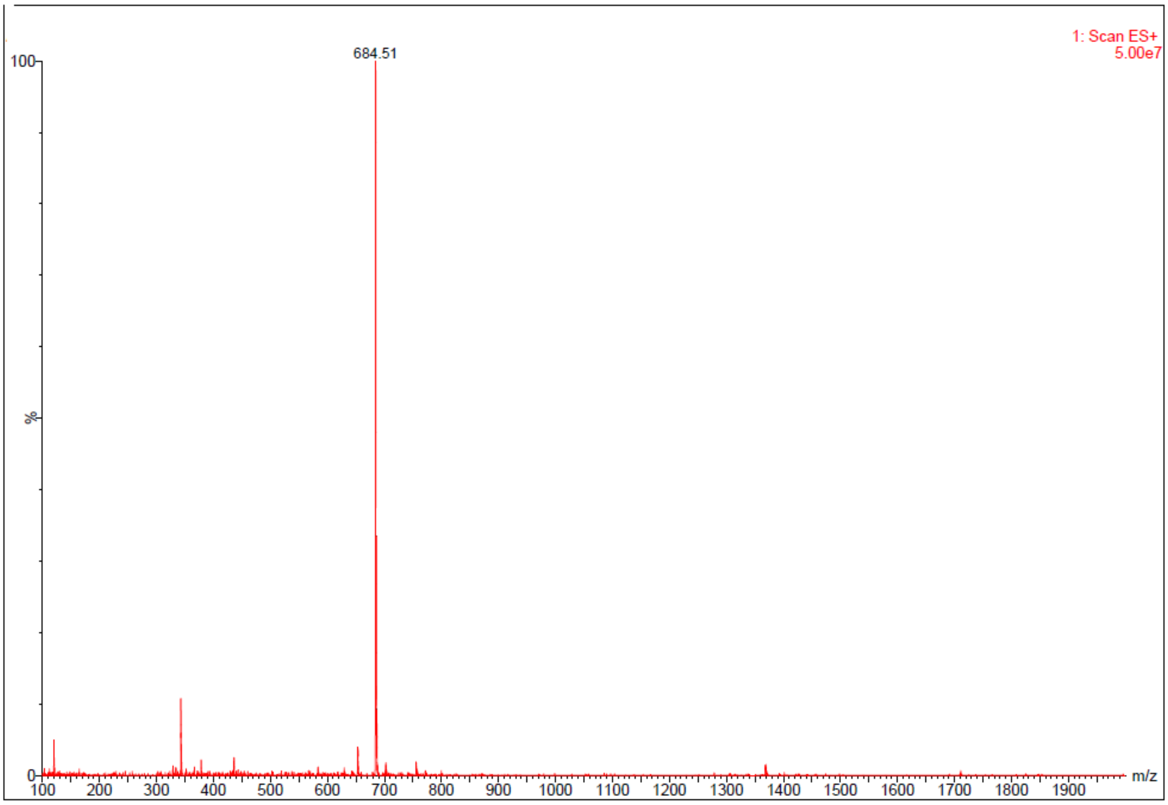

Figure S15: MS (ESI+) spectrum of compound **8**.

Molecular modelling simulations of conjugate 9.

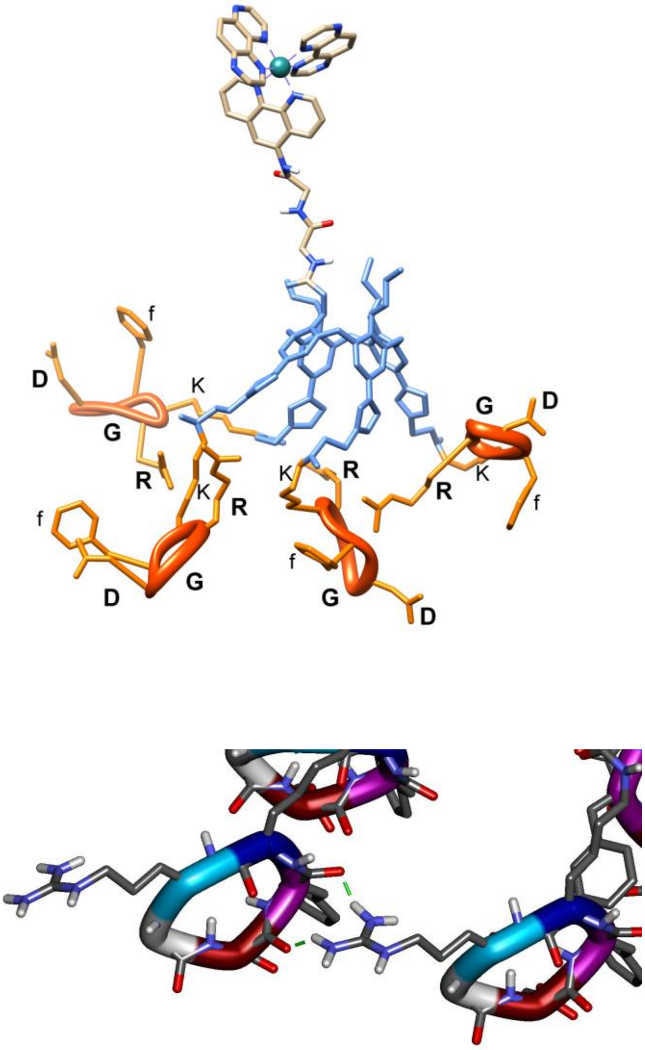

Figure S16: MD snapshot showing an optimized model of conjugate **9**. RGDfK units are depicted in orange ribbons, the calixarene in blue and the Ru complex coloured by atom type. Bottom: zoom of a snapshot showing typical H-bonds between neighboring RGDfK units (only polar H atoms are shown); the cyclic peptides are depicted as tubes colored by residue.

Quenching studies on conjugate 7.

Luminescence quenching studies were performed on conjugate **7** in the presence of the free cyclic pentapeptide units c-[RGDfK] **8** in order to evaluate the possible occurrence of an internal quenching process in conjugate **9** between the ruthenium centre and the c-[RGDfK] moieties.

Increasing concentrations (0, 0.5 and 1 mM) of c-[RGDFK] peptide **8** were added to a solution of $[Ru(TAP)_2phenGG]^{2+}$-$X_4Pr_3(N_3)_4$ **7** (10^{-5}M).

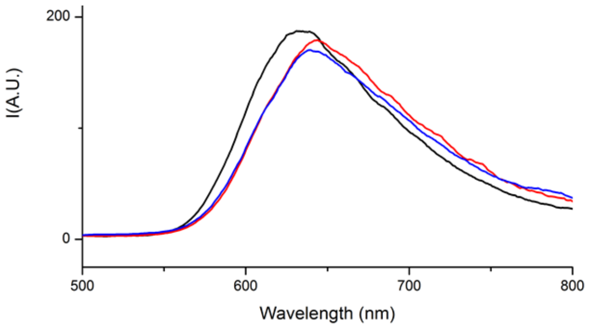

Figure S17: Emission spectrum of **7** (10^{-5}M) (in black) and in the presence of 0.5 and 1 mM of **8** (in blue and red respectively). Measured under air at room temperature in acetonitrile.

As depicted in Figure S17, the presence of the free cyclic pentapeptide units c-[RGDfK] **8** does not have a significative influence on the luminescence intensity of the intermediate **7**. Determination of the luminescence lifetime in the same conditions, *i.e.* for **7** alone in MeCN under air (625 ns) and for **7** in the presence of 0.5 and 1.0 mM of c-[RGDfK] **8** (644 and 692 ns respectively), confirms that no quenching is taking place between the ruthenium complex and RGD peptidic units.

In conclusion, we can safely assert that no internal quenching process is taking place in conjugate **9** and that the photophysical and photochemical properties of the ruthenium complex anchored on the calixarene platform are conserved.

References

[1] Degardin, M.; Thakar, D.; Claron, M.; Richter, R.P.; Coche-Guérente, L.; Boturyn, D. *J. Mater. Chem. B* **2017**, *5*, 4745-4753.
